# Trash‐to‐Energy: Shedding Light on Plastic and Biomass Valorization Through Artificial Photosynthesis Towards Sustainability

**DOI:** 10.1002/EXP.20240344

**Published:** 2025-08-10

**Authors:** Ke Ming Lim, Valerine Khoo, Wee‐Jun Ong

**Affiliations:** ^1^ School of Energy and Chemical Engineering Xiamen University Malaysia Selangor Darul Ehsan Malaysia; ^2^ Center of Excellence for NaNo Energy & Catalysis Technology (CONNECT) Xiamen University Malaysia Selangor Darul Ehsan Malaysia; ^3^ State Key Laboratory of Physical Chemistry of Solid Surfaces College of Chemistry and Chemical Engineering Xiamen University Xiamen China; ^4^ Gulei Innovation Institute Xiamen University Zhangzhou China; ^5^ Shenzhen Research Institute of Xiamen University Shenzhen China; ^6^ Department of Chemical and Biological Engineering College of Engineering Korea University Seongbuk‐gu Seoul Republic of Korea

**Keywords:** clean fuels, photocatalysis, solid waste upcycling

## Abstract

Solid waste remains a global crisis in which massive amount of solid waste is disposed of in landfills and the environment yearly, leading to detrimental environmental pollution and loss of resources. However, the current downcycling technologies, such as pyrolysis and gasification, usually require extensive energy input and harsh operating conditions, posing a high possibility of causing secondary pollution. In pursuit of a sustainable future, artificial photosynthesis arises as one of the promising but arduous approaches to reform solid waste into fuels and commodity chemicals under benign conditions. Under this backdrop, this review aims to present a thorough overview of the recent advancement in solid waste transformation through photocatalysis. To begin with, the principles of solar‐driven conversion pathways for solid waste are discussed under different reaction conditions. Then this review also highlights the merits of artificial photosynthesis and diverse state‐of‐the‐art photocatalysts for solid waste valorization. Special emphasis is placed on elucidating the application of external‐field‐assisted photocatalysis (e.g. photothermocatalysis, photoelectrocatalysis, photobiocatalysis, and piezo‐photocatalysis) for solid waste upcycling to explore the synergistic effects on performance improvement. Finally, insights on the challenges and prospects in photocatalytic solid waste conversion are presented to bridge a new exemplification towards a sustainable circular economy in the future.

## Graphical abstract

To engrave a circular economy, artificial photosynthesis emerges as a promising and prosperous strategy to upcycle solid waste into clean fuel and valuable chemicals for sustainable fuel production. This review highlights the design of photocatalysts and external‐field‐assisted photocatalysis for effective solid waste conversion toward an environmentally friendly and sustainable future.

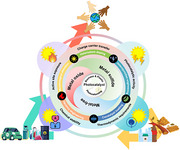

## 1 Introduction

The rapid development of society and growing global population have caused exponentially increasing demand for energy, while most energy resources (e.g. natural gas, petroleum, and coal) used in daily life have been depleting over the years, resulting in the energy shortage and environmental degradation. Although renewable energies, such as solar, wind, tide power, and geothermal, have become more vital in electricity generation for anthropogenic activity, fossil fuels still contributed 80% of global energy demand in 2023 [1]. Transportation, industrial activity, and power generation account for most carbon emissions by burning carbon‐based fuels, reaching a critical level in the atmosphere. For instance, in December 2024, the CO_2_ atmospheric concentration surged to 427.85 ppm compared to December 2023 (421.66 ppm) based on the Mauna Loa Station [2]. Hence, along with this energy crisis and accelerating environmental issues, the conventional linear “take‐produce‐use‐dispose” economic model that has been used for a couple of decades is expected to be replaced by the circular economic model for prioritizing future sustainability. At this moment, revolutionizing the use and production of resources and energy is promptly desirable for the transition toward a circular economy. Storing the energy of sunlight as solar fuels (e.g. H_2_ and hydrocarbons) can play a crucial role in bridging the gap to contribute to a sustainable energy system, whereas solar fuels have a wide range of application from clean transport fuels, heating, and synthesis of chemicals [3].

On the other hand, solid waste is another major contributor to greenhouse gas emissions as over a billion tons of solid waste are yielded worldwide annually, which more than 50% suffer from improper and ineffective waste management, including landfills, open dumps, open burning etc. [4, 5]. Taking plastic waste as an example, there are over 8 billion tons of plastics produced worldwide up to now of which around 80% have been discarded via landfill or directly to nature, resulting in “white pollution” [6, 7, 8]. Despite promoting the 3Rs (i.e. reduce, reuse, and recycle) in society and enhancing industrial technologies to address the solid waste crisis, discovering the worth in solid waste that cannot be repurposed is also essential to prevent the depletion of potential sustainable resources. In recent years, several studies have shown that the plentiful solid waste produced from anthropogenic activity —such as plastic and biomass— can be transformed into high‐value chemicals using green technology, thereby helping to mitigate the waste pollution crisis [9, 10, 11, 12, 13, 14]. Under this backdrop, it is imperative to explore alternative carbon‐rich energy sources, such as plastic and biomass, and integrate them with abundant solar energy for developing advanced green technology to secure a harmonious and resilient future.

**FIGURE 1:**
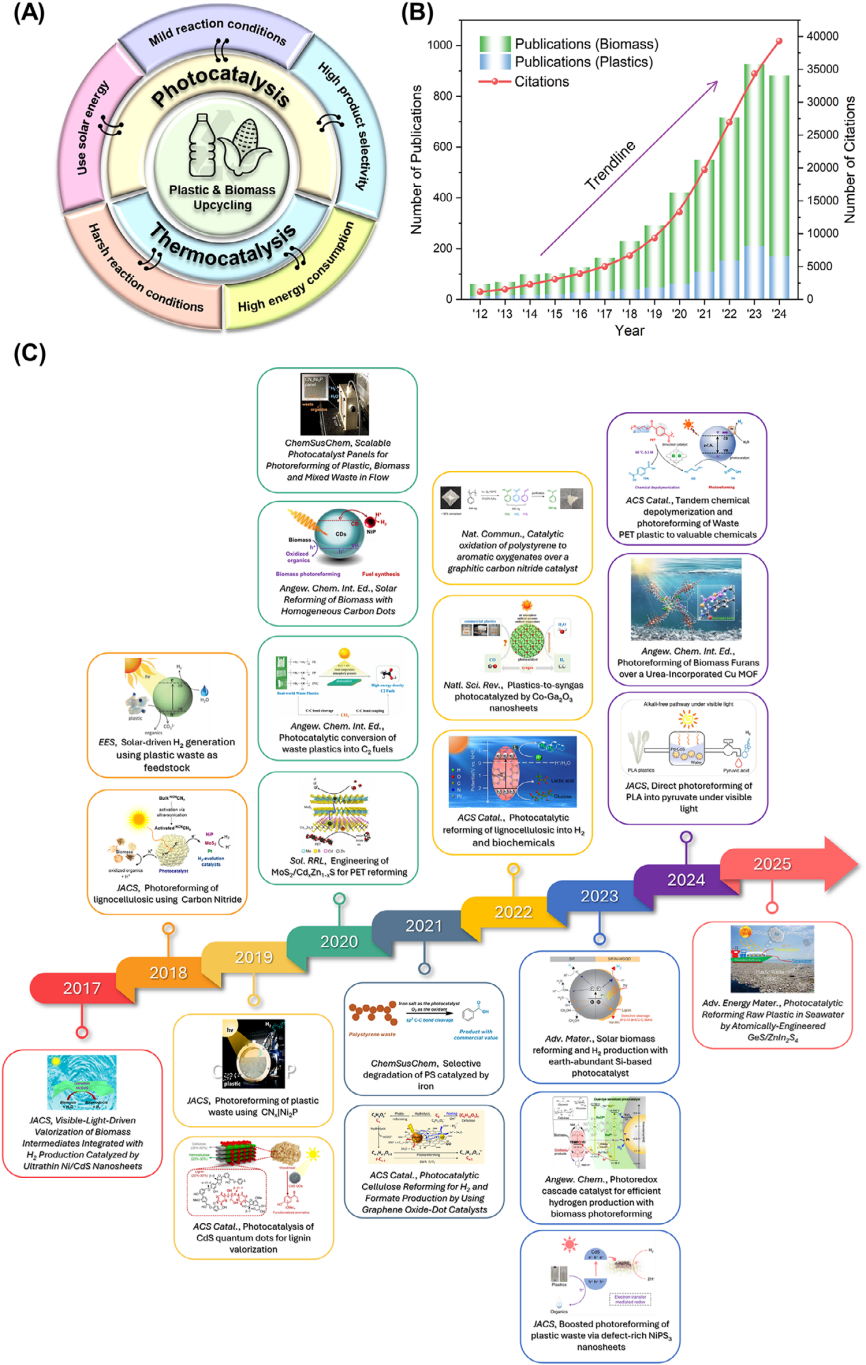
(A) Overview of comparison between catalytic upcycling methods for solid waste via photocatalysis and thermocatalysis. (B) Number of annual publications and citations with the topic keywords “photocataly*,” “plastic,” and “biomass” since 2012, sourced from Web of Science database, dated 19th December 2022. (C) Timeline of recent advancement in photocatalytic reforming of solid waste since 2017 [47, 48, 49, 50, 51, 52, 53, 54, 55, 56, 57, 58, 59, 60, 61, 62, 63, 64, 65, 66]. Reproduced with the permission of Kasap et al. [47] Copyright 2018, American Chemical Society. Reproduced under the terms of the Creative Commons CC BY license [48]. Copyright 2019, American Chemical Society. Reproduced with the permission of Wu et al. [49] Copyright 2019, American Chemical Society. Reproduced under the terms of the Creative Commons CC BY license [50]. Copyright 2022, Oxford University Press. Reproduced with the permission of Wang et al. [51] Copyright 2022, American Chemical Society Reproduced with the permission of Zhang et al. [52] Copyright 2023, American Chemical Society. Reproduced under the terms of the Creative Commons CC BY license [53]. Copyright 2023, Wiley‐VCH. Reproduced with the permission of A. Kobayashi [54] Copyright 2023, Wiley‐VCH. Reproduced under the terms of the Creative Commons CC BY license [55]. Copyright 2024, Wiley‐VCH. Reproduced with the permission of Li et al. [56] Copyright 2024, American Chemical Society. Reproduced with the permission of Miao et al. [57] Copyright 2024, American Chemical Society. Reproduced with the permission of Li et al. [58] Copyright 2021, Wiley‐VCH. Reproduced with the permission of Uekert et al. [59] Copyright 2018, Royal Society of Chemistry. Reproduced with the permission of Wang et al. [60] Copyright 2021, Wiley‐VCH. Reproduced with the permission of Jiao et al. [61] Copyright 2020, Wiley‐VCH. Reproduced with the permission of Talebian‐Kiakalaieh et al. [62] Copyright 2025, Wiley‐VCH. Reproduced with the permission of Han et al. [63] Copyright 2017, American Chemical Society. Reproduced with the permission of Nguyen et al. [64] Copyright 2017, American Chemical Society. Reproduced under the terms of the Creative Commons CC BY license [65]. Copyright 2022, Springer Nature. Reproduced with the permission of Uekert et al. [66] Copyright 2020, Wiley‐VCH.

In light of the natural photosynthesis process that occurs in plants, artificial photosynthesis keeps the promise distinctly to generate fuels and chemicals by using sunlight with the aid of multitudinous theoretical and experimental studies over the decades. Unlike low solar‐to‐biomass conversion efficiency (1‐2% for crops) from biological photosynthesis process, various energy conversion reactions, including H_2_O splitting, CO_2_ reduction, and N_2_ fixation, in artificial photosynthesis can be applied via semiconductor with escalating solar‐to‐chemical conversion efficiency [15, 16, 17, 18, 19, 20, 21, 22, 23, 24, 25, 26]. In particular, photocatalytic H_2_O splitting reactions that consist of hydrogen evolution reaction (HER) and oxygen evolution reaction (OER) are always limited by the latter reaction due to the sluggish kinetics and high thermodynamic barriers. On top of that, these solar energy conversion reactions in the previous studies are focused on the reduction cycles rather than oxidation cycles, leading to a scarcely detailed discussion of oxidation half‐reaction [27, 28, 29, 30]. Different from introducing typical sacrificial reagents (e.g. triethanolamine, methanol, lactic acid, and Na_2_S), the oxidation of organics that is more kinetically favorable can replace OER to consume photoinduced holes to improve the overall efficiency of the photocatalytic reaction, as evidenced by the coupling of HER and organic transformation [22, 24, 31, 32, 33]. In these circumstances, plastic and biomass from solid waste can be considered as waste polymers, which are composed of a long chain of organic molecules, to serve as a versatile feedstock for further conversion. Typically, the current development of plastic and biomass treatment technologies usually featured mechanical and chemical recycling, thermal treatment (e.g. incineration, liquefaction, and pyrolysis), and biochemical breakdown (e.g. composting, fermentation, and anaerobic digestion) to downcycle or reprocess the waste into new products [34, 35, 36]. Currently, upcycling catalytic methods for solid waste valorization, especially photocatalysis, have been put under the spotlight to produce high‐value products with additional economic merits and simultaneously reduce the use of mechanical recycling, incineration, and landfills [37, 38, 39, 40, 41, 42, 43]. Photocatalysis has been deemed as a cheaper and greener method due to its benign conditions of ambient temperature and pressure utilizing sunlight as an energy source [44]. The mild conditions applied in photocatalysis possess great potential to realize the selective generation of target products by precisely activating certain chemical bonds with other functional groups. Solar‐driven solid waste transformation emerges as a promising and innovative approach for simultaneous solar fuels generation and solid waste oxidation to address energy challenges and contemporary environmental issues for achieving a more sustainable and eco‐friendly future. On the contrary, the thermochemical route, such as gasification and pyrolysis, usually requires harsh reaction conditions, large energy inputs, and extensive use of metal catalysts, which is not beneficial for sustainable development with debatable long‐term feasibility (Figure 1A).

Due to its mild conditions and generation of value‐added fuels, and chemicals, the solid waste upcycling via photocatalysis has received explosively increasing research interest in the last 10 years, which was undoubtedly proven by the search using the term “photocataly*” “plastic”, and “biomass” as the keywords in Web of Science database (Figure 1B,C). Recently, several reviews regarding photocatalytic conversion of solid waste have been published with the fundamental comprehension of catalytic upcycling of solid waste. For example, Feng and his team summarized the transformation of biomass and plastic into valuable renewable chemicals under mild conditions by heterogeneous photocatalysis to realize photoredox reaction [45]. Furthermore, Chu et al. reviewed the recent advancement in plastic upgrading using artificial photosynthesis, with a concern on the selective transformation of plastics via bond cleavage [44]. Lieu et al. systematically reviewed the plastic waste conversion by different catalytic technologies, including photocatalysis, electrocatalysis, and photoelectrocatalysis, yet limited emphasis has been put on other external‐field‐assisted processes [46]. Unfortunately, there is a lack of understanding on the design of photocatalysts with the presence of external‐field‐assisted process for solar‐driven solid waste transformation in previous reviews. In view of the significance of photosynthetic upcycling of solid wastes, a critical review of the latest progress on various photocatalysts in solid waste photoreforming is a necessity to provide some insights into the design of high efficiency photocatalytic system for solid waste valorization. This review begins with the mechanism of photocatalytic waste conversion system and the advantages of solid waste photoreforming. Next, the utilization of different types of photocatalyst in photoreforming is discussed in detail to provide an in‐depth understanding of the modification strategies and performance of the photocatalyst. Moving on, the application of external‐field‐assisted process in photoreforming is highlighted, specifically thermal, biocatalysis, piezoelectric and electrochemical. Last but not least, this review provides a summary and insights into the future perspectives and challenges of solid waste photoreforming.

## 2 Photocatalytic Solid Waste Conversion Pathways

As a vast available feedstock in artificial photosynthesis, solid waste generally can be divided into two main types including plastic and biomass. The first artificial polymer material in the world—bakelite—was synthesized by Leo Baekeland in 1907 [67]. Owing to the benefits of low cost, safeness, processibility, and mass production, a variety of polymers, such as polyethylene (PE), polypropylene (PP), polyethylene terephthalate (PET), polystyrene (PS), polyvinyl chloride (PVC) etc., has been progressively manufactured and become the material of choices for the domain of electronic devices, packaging, households, and surgical goods [68]. As an indispensable commodity in human daily lives, polymer materials, commonly known as plastics, have had increasing demand over the years, leading to a rise of 9% in plastic production per annum. Heretofore, the global production of plastics reached approximately 360 tons in 2018 and is anticipated to exceed 5000 million tons by 2050 [6, 69, 70]. Miserably, the waste treatment technologies were not enhanced concurrently to correspond to the production rate of plastics, resulting in more and more plastic waste in the environment [71]. The plastic waste generally takes centuries to degrade spontaneously, resulting in continual amassing in nature. Landfill and direct disposal of waste to the environment not only hinder sustainable land use but also invade the food web and cause contamination to crops, animals, aquatic organisms, and humans, threatening the whole ecosystem [72]. Of note, plastics can be classified into two types, including polar and non‐polar plastics. Polar plastics, for example, PET, polyurethane (PUR), and polylactic acid (PLA), possess plentiful oxygen‐containing functional groups, which can readily hydrolyze into monomers under alkaline conditions [73]. Hereby, valuable chemicals, such as formic acid, acetic acid, and methanol, can be acquired through solid waste photosynthesis in such an environment. Distinct from polar plastics, non‐polar plastics such as PE and PP are difficult to break down in nature due to the hydrophobicity and the stable C─C bond in the long hydrocarbon backbone.

A typical product generated from natural photosynthesis, biomass, is the most abundant and readily accessible natural organic material, with 170 billion tons of production per year [74]. Gratifyingly, biomass has been considered as a potential alternative to substitute fossil fuel for a sustainable clean energy supply, as evidenced by the successful production of bio‐based chemicals (e.g. lactic acid, succinic acid, glycerol etc.) [75]. In general, lignocellulosic biomass consists of three main constituents: 30–50% cellulose, 25–30% hemicellulose, and 15–25% lignin, where the former two components were usually encapsulated by lignin [76, 77, 78]. Typically, cellulose is a polysaccharide polymer composed of d‐glucose and cellobiose by β‐1,4‐glycosidic bond [79], while hemicellulose is another long chain polymer consisting of randomly branched molecules such as xylose, mannose, glucose, galactose, uranic acids, fucose, rhamnose, and arabinose [80]. In contrast to cellulose and hemicellulose, lignin has a more complex structure due to the nature of monomers and irregular bonding [81]. Particularly, lignin is mainly made up of three aromatic allylic alcohols, including coniferyl alcohol, sinapyl alcohol, and paracoumaryl alcohol, whereby these monomers are connected by C─O (β‐O‐4, α‐O‐4 etc.), and C─C linkages (*β*‐1, *β*‐5 etc.) [82, 83]. Such stable crosslinking and complex structures endow lignin rigidity and recalcitrance, hence leaving a challenge to convert biomass into small fragments. The lignin portion varies depending on the type of biomass, yet softwood usually has higher lignin content than hardwood and agricultural waste [84, 85]. Given the large availability of plastic and biomass worldwide, developing an efficient and sustainable technology to take advantage of such energy resources thoroughly is highly desirable for the carbon and waste circular economy.

These waste polymers with rigid chemical structures usually require pretreatment for the realization of selectivity in photoreforming. Of note, the pretreatments have the ultimate significance of acquiring more available monomers for photoreforming process through the alteration of polymer structure. Such pretreatment consists of physical, chemical, physicochemical, and biological approaches. An effective pretreatment method for waste photoreforming should always consider several aspects: (i) cost‐effective operation, (ii) flexible scalability towards industrial application, and (iii) high yield of monomer substrate to produce value‐added products and clean fuels. In current studies, chemical pretreatment (i.e. acid and alkali) is commonly used to break down the persistent structure of plastic and biomass [51, 59, 86]. Albeit these two pretreatment methods facilitate the depolymerization of solid waste, they are not viable for a larger scale owing to the cost and ecological effects of acid and base. On the other hand, the physical treatment, including mechanical fragmentation (e.g. pulverization and ball milling) and plasma, aims to release the intermediate components by diminishing the particle size and increasing the exposed surface area [50, 87, 88]. In fact, mechanical processes usually have a higher feasibility for practical application, yet the controversy of this technique lies in the increment of surface area without affecting the structural properties of plastic and biomass. In contrast, the plasma‐assisted process can reduce the activation energy barrier, shorten the reaction duration, and accelerate the monomerization/product formation [89]. The challenges of this technology remain in large‐scale applications due to the high operational cost and shortage of efficient microwave absorbers and high‐capacity equipment.

Generally speaking, the physicochemical method involves the use of chemicals and reaction conditions to process the waste polymers. By using the hydrothermal method, plastic waste and biomass can be hydrolyzed in the presence of a certain amount of acid and subcritical water [57, 90]. Nevertheless, the use of high temperatures to fracture the solid waste and the large energy requirement are contradictory to green chemistry only if the energy is acquired from renewable sources. Instead of using heat and chemicals to treat the waste polymers, biocatalysis that involves microbial enzymes offers an energy‐free method with promising scalability for depolymerization. For example, the hemicellulose can be degraded using various enzymes, including *α*‐glucuronidases, acetyl xylan esterases, endo‐1,4‐*β*‐xylanases, and *β*‐d‐xylosidases [91]. This bioprocessing technology still suffers from the high cost of cultivating different enzymes, limited substrate types for enzymatic degradation, and a time‐consuming process (i.e. lengthy incubation time and slow reaction rate) [92, 93]. With these contexts, different factors govern the selection of pretreatment methods for photoreforming process, including the type of waste, the cost of pretreatment, product selectivity, and product state. All in all, the in‐depth analysis from the perspective of economics and sustainability is necessary to explore the ideal pretreatment methods for industrial processes.

### 2.1 Arising Solar‐Driven Pathways: Nonselective Degradation vs. Selective Synthesis

**FIGURE 2:**
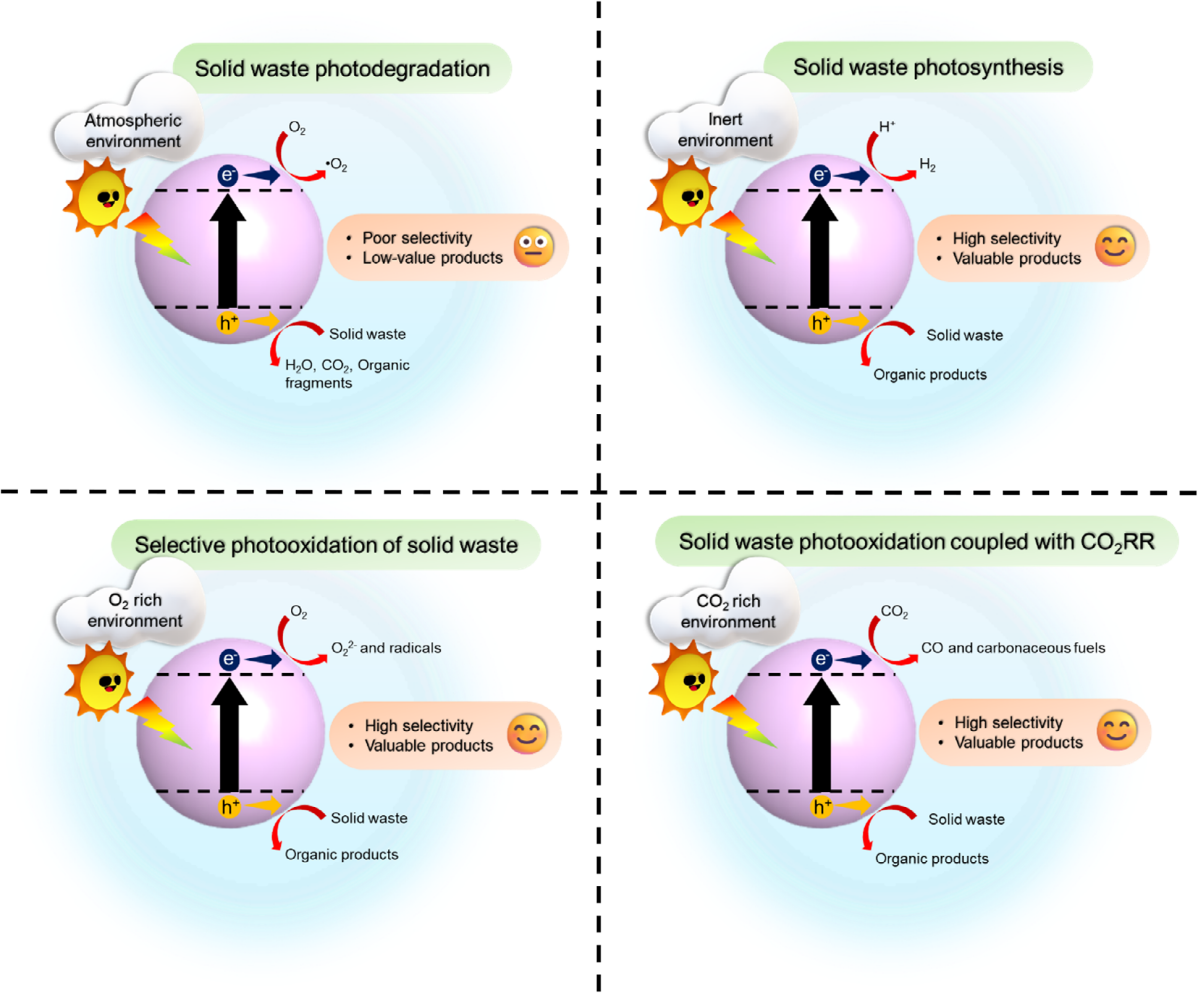
Differences between the solid waste photocatalytic transformation system.

The exploitation of solar energy in the waste conversion system offers an environmentally friendly and inexpensive method to valorize solid wastes for fuels and useful chemical products. The solar‐driven catalytic process can not only aid in plastic and biomass degradation in the environment but also generate value‐added chemicals by initiating a redox reaction, which is analogous to the concept of “Waste to Wealth.” For example, Zhu et al. prepared a copper phthalocyanine‐modified TiO_2_ to realize the degradation of polystyrene to CO_2_ [94]. Meanwhile, Hou and coworkers realized the photocatalytic conversion of lignin β‐1 model to benzaldehyde over a CuO*_x_*/ceria/anatase nanotube hybrid [95]. As such, photocatalytic waste conversion systems can be classified into two types: photodegradation and photosynthesis (waste photoreforming, selective photooxidation, and carbon dioxide reduction coupled with photooxidation) (Figure 2). The major difference between them is that solid waste is treated to be mineralized into CO_2_ molecules in the photodegradation process, while photosynthesis places an emphasis on employing plastic wastes and biomass as alternative resources to generate recoverable and high‐value chemicals [44]. Therefore, the selective generation of target products is the key criterion for differentiating photosynthesis from photodegradation. Moreover, atmospheric condition (i.e. in the presence of air) is usually employed in the photodegradation process to produce highly oxidizing species such as hydroxyl radicals, leading to nonselective oxidation reactions [96, 97]. These strong oxidizing agents may destroy solid waste rather than precisely transform it into value‐added organics. In photosynthesis, the anaerobic or specific condition is commonly applied, and the photoinduced holes in the valence band of the semiconductor are used to promote selective solid waste transformation into value‐added products; at the same time, the photogenerated electrons would reduce protons to yield high purity of H_2_ or other solar fuels to achieve photoredox reaction. Particularly, the generation of aromatic oxygenates and gluconic acid is realized through the conversion of polystyrene and glucose under an O_2_ atmosphere owing to the abundance of superoxide anions.

#### 2.1.1 Photodegradation Process & Mechanism

Considering that the solid wastes are recalcitrant and pernicious pollutants to the environment, it is essential to take immediate action to eradicate them. Distinct from other strategies such as gasification and incineration, the photodegradation process is an environmentally friendly and feasible method to eliminate solid waste. It can directly make use of the abundant solar energy to convert solid wastes into water and CO_2_ under mild conditions of ambient temperature and pressure [72]. Besides, since plastic wastes and biomass are often exposed to nature, they can readily undergo solid‐phase photodegradation under the irradiation of sunlight [98]. In contrast to the liquid‐phase system with the solid wastes and photocatalysts suspended in solution form, there are a good deal of chances of reactive oxygen species (ROS) and holes that can react with plastics and biomass in the solid phase owing to the direct and close contact between the solid wastes and photocatalyst without any impediment [44]. Typically, the degradation of plastics wastes first initiates at the interface between the photocatalyst and plastics, and subsequently extends to the plastic matrix via the diffusion of ROS generated on the surface of the photocatalyst [99, 100, 101].

Of note, the photodegradation mechanism of plastic wastes is also similar to the photocatalytic oxidation of other organic substances, such as lignocellulosic biomass [102]. When the photocatalysts absorb sunlight with higher photon energy than the band gap energy, the photoexcited electrons are transferred or shifted into the conduction band (CB), initiating the reduction of O_2_ to superoxide anion radicals (O_2_ **^−^**). Then, these superoxide ions can further undergo a series of reactions to generate the highly active hydroxyl radicals (**·**OH). Meantime, the photogenerated holes (h^+^) located in the valence band (VB) would move to the surface of the photocatalyst, wherein the holes can directly oxidize the solid wastes or react with adsorbed H_2_O to produce more **·**OH and degrade or mineralize the solid wastes into multifarious small molecular organic compounds or ultimately to H_2_O and CO_2_ [40, 103]. In the course of the photodegradation process, the ROS are nonselective, thus resulting in the formation of a complex mixture of products such as small organic fragments, mineralized products, microplastics, and nanoplastics. Fundamentally, the generation of reactive oxygen species and the photodegradation process of plastic wastes are represented below:

(1) $$\mathrm{Photocatalyst}+\mathrm{photon}\to e^{-}+h^{+}$$

(2) $$h^{+}+H_{2}O\to \cdot \mathrm{OH}+H^{+}$$

(3) $$O_{2}+e^{-}\to {\cdot O}_{2}^{-}$$

(4) $$2H^{+}+\cdot O_{2}^{-}+e^{-}\to H_{2}O_{2}$$

(5) $$H_{2}O_{2}+\mathrm{photon}\to 2\cdot \mathrm{OH}$$

(6) $$\cdot {\mathrm{OH}\;\mathrm{or}\;O}_{2}^{-}+\mathrm{solid}\;\mathrm{waste}\;\left(C_{x}H_{y}O_{z}\right)\to {\mathrm{CO}}_{2}+\mathrm{organic}\;\mathrm{fragments}$$

**FIGURE 3:**
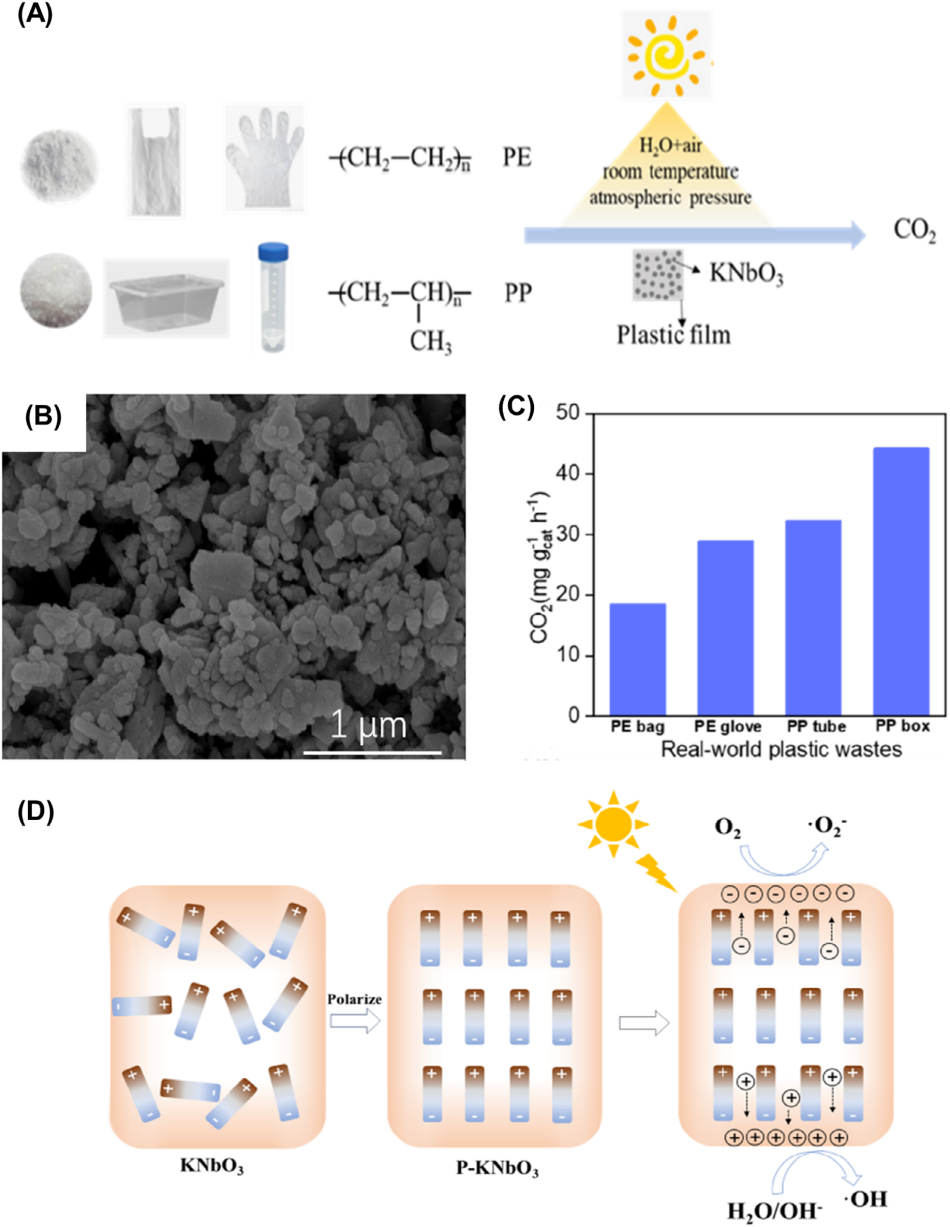
(A) Scheme of conversion of PE and PP into CO_2_. (B) TEM image of P‐KNbO_3_ nanosheet. (C) Production of CO_2_ during the photocatalytic conversion of real‐world plastic bag, glove, tube, and box over P‐KNbO_3_. (D) Scheme of photogenerated charge transfers in P‐KNbO_3_ upon illumination [107]. Reproduced with the permission of Liu et al. [107] Copyright 2022, Elsevier.

Among different types of plastics, polyolefins such as polyethylene (PE) and polypropylene (PP), which constitute 57% of total plastic production, are the most broadly researched in photodegradation studies [104, 105, 106]. Taking PE and PP as the examples, Liu et al. recently reported a polarized ferroelectric KNbO_3_ (P‐KNbO_3_) photocatalyst for plastics degradation under mild conditions (Figure 3A,B) [107]. P‐KNbO_3_ photocatalyst resulted in excellent CO_2_ production rates for the photodegradation of PE and PP owing to the enhancement from polarization treatment to the photocatalytic ability of KNbO_3_ (Figure 3C). The PE and PP were eventually mineralized into CO_2_ due to the attack of generated h^+^, **·**OH, and **·**O_2_^ˉ^ towards C─H bonds (Figure 3D). Apart from plastics, the photodegradation of biomass has also been widely studied. For instance, the degradation of lignin over titania photocatalyst was first realized by Kobayakawa et al. in 1989 [108]. Overall, the photodegradation process is an eco‐friendly and effective way to eliminate solid wastes by converting them into CO_2_ and H_2_O. Nevertheless, the end product (CO_2_) and other possible intermediates have negative or hidden effects on the environment. Particularly, the production of CO_2_ gas could contribute to the emission of greenhouse gases. Additionally, only a few merits have emerged in the photodegradation process since biomass and plastic are treated as wastes that need to be eliminated. Under this context, the utilization of carbon‐ and hydrogen‐rich biomass and plastics as chemical resources to photosynthesize them into valuable products is a more attractive route from the perspective of economics and sustainability.

#### 2.1.2 Photocatalytic Upcycling of Solid Waste

**FIGURE 4:**
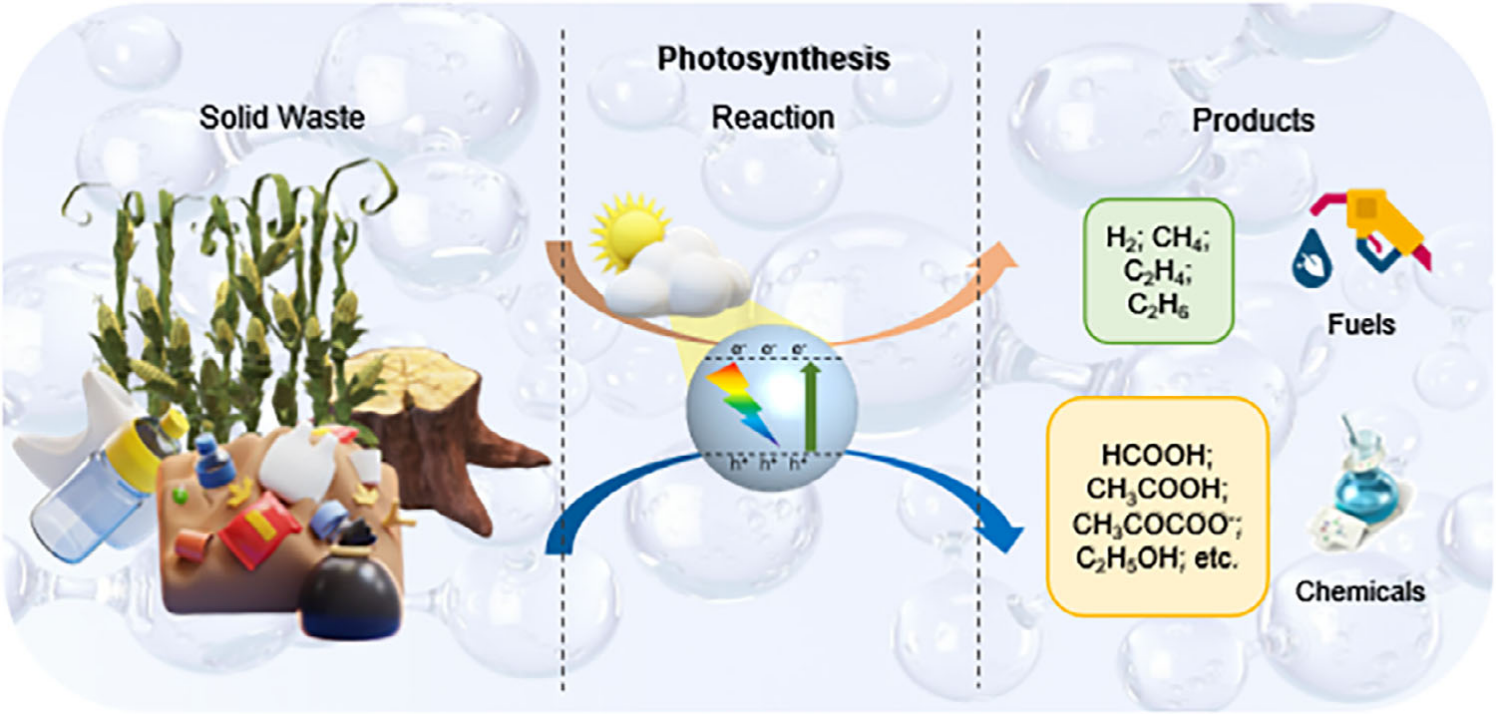
Scheme of photosynthesis from solid wastes into fuels, chemicals, and materials.

The photosynthetic upcycling of solid wastes can not only diminish the waste pollution but also generate value‐added chemicals. Among the other upcycling methods, photocatalytic upgrading is an environmentally benign and promising technique that treats solid wastes as a chemical resource to yield valuable products. In contrast with the few advantages brought by photodegradation, photosynthesis has a higher atom economy and outstanding economic superiority, which is in agreement with the concept of environmental sustainability [44]. Hitherto, recent studies have focused on the photosynthesis of solid wastes, including cellulose, lignin, PE, PP, and PS, into fuels (i.e. H_2_), and chemicals (i.e. formic acid, acetic acid, pyruvate, ethanol, ethane, propane), as illustrated in Figure 4.

The photosynthesis of solid wastes for H_2_ production, also known as photoreforming, has a similar mechanism to that of common photocatalytic H_2_ evolution utilizing organic electron donors [109]. During the solid waste photoreforming process, the plastics or biomass serve as sacrificial agents to be oxidized by photoinduced holes, while the photogenerated electrons reduce water to generate H_2_. In terms of the thermodynamic viewpoint for solid waste photoreforming, the CB potential of the photocatalysts should be more negative than the potential for H^+^ reduction to H_2_ (0 V vs. reversible hydrogen electrode, RHE), whereby the photogenerated electrons are able to reduce water to H_2_. Meanwhile, the potential of VB in the photocatalysts should be more positive than the potential for solid waste oxidation (around 0 V vs. RHE), thus transforming the wastes into valuable chemicals [44, 110]. Under solar irradiation, the electrons in the photocatalyst are excited and migrate to the CB, which then reduces protons to yield H_2_. The photogenerated holes on the VB would get involved in the oxidation of substrates via direct or indirect pathways [44].

The direct pathway incorporates the hole transfer to oxidize the biomass and plastic wastes without the intermediary of oxidizing species. On the other hand, the indirect pathway takes advantage of hydroxyl radicals generated from the reaction of holes with H_2_O to oxidize the biomass and plastic wastes indirectly [44]. The reaction pathways that occur in photoreforming are usually affected by several factors, such as substrate, photocatalyst, ROS, and reaction conditions. For instance, TiO_2_ and ZnO photocatalysts generally can react based on the indirect pathway because they have a higher VB position in comparison with the potential of the **·**OH/H_2_O redox pair (+2.27 V vs. RHE). Whereas, photocatalysts such as CdS and MoS_2_ that have inadequate photooxidation ability are likely reacting according to the direct pathway [103]. Despite the nature of both reaction pathways, the photoreforming of solid wastes often produces a complex mixture containing products such as organic compounds and CO_2_. In general, a brief expression of the photoreforming process of solid wastes is shown below:

(7) $$\mathrm{Photocatalyst}+\mathrm{photon}\to e^{-}+h^{+}$$

(8) $$h^{+}+H_{2}O\to H^{+}+\cdot \mathrm{OH}$$

(9) $$2e^{-}+2H^{+}\to H_{2}$$

(10) $$h^{+}\;\mathrm{or}\;\cdot \mathrm{OH}+C_{x}H_{y}O_{z}\to \text{Organic products}+{\mathrm{CO}}_{2}$$

Whilst photoreforming studies usually focus on the hydrogen evolution reaction (HER) from H^+^ reduction, the photosynthesis from solid wastes into chemicals mainly pays attention to the solid waste oxidation for the formation of desirable carbonaceous products or fuels. These carbon‐containing compounds can be acquired either through direct oxidation of waste via hole transfer or an indirect pathway by ROS [44]. The critical challenge in this technique is to achieve high selectivity towards satisfactory products and impede the formation of unfavorable by‐products. Thereby, the formation of nonselective highly oxidizing species should be prevented to improve the production rate and selectivity of desirable products. Beyond the single‐step photocatalytic reaction, the carbon‐based products can also be obtained by a two‐step route, such as plastics photooxidation into CO_2_ through C─C bond scission, followed by CO_2_ photoreduction per C─C bond coupling [44]. Additionally, as exemplified by the work of Zhang and his group, a binuclear Re‐Ru complex was prepared for plastic waste upcycling coupled with CO_2_ photoreduction to generate syngas (i.e. CO and H_2_) and valuable chemicals [111].

Aside from converting the solid wastes into fuels and chemicals, the postsynthetic modification of plastics into materials has also attracted growing interest from the research community. The functional group transformation of plastics usually refers to C─H functionalization in plastics. The C─H functionalization in plastics has a similar photocatalytic mechanism to the C─H bond photoactivation of small organic compounds [112, 113]. Generally, the formation of alkyl radicals from the C─H bond cleavage is deemed the rate‐limiting step for the functionalization of plastic. The photoinduced holes and ROS are the necessary active species for C─H bond cleavage in the photoactivation process of C─H bond.

Nonetheless, the byproducts with environmental impacts can be generated while monomerizing the long‐chain polymers through the pretreatment and photocatalysis. Plastic (e.g. PS and PET) and biomass (i.e. lignin) contain many aromatic rings in their chemical structure, in which these materials tend to break down into aromatic hydrocarbons, such as benzene and toluene [114]. These persistent aromatic components are toxic to organisms, leading to carcinogenic effects on the human body. On the other hand, the complete and partial oxidation of solid waste can lead to the production of CO_2_ and CO gases, respectively, which can contribute to climate change through greenhouse gas emissions. To mitigate the environmental risks of these byproducts, several techniques, including life‐cycle assessment (LCA), ecological risk assessment (ERA), and environmental impact assessment (EIA), are vital to employ for identifying, managing, and reducing potential environmental footprints on the ecosystem [115, 116, 117]. Next, developing selective photocatalysts is also desirable to promote a clean transformation of solid waste to value‐added chemicals. In short, using LCA, EIA, and ERA can pave the way for developing a cleaner and circular waste economy, ensuring that the waste photoreforming technology is efficient, sustainable, and safe upon utilization.

### 2.2 Advantages towards photoreforming of solid waste

In the last couple of years, photocatalytic water splitting, which consists of the two half‐reactions (i.e. OER and HER), has been widely studied for clean H_2_ fuel production [118, 119, 120, 121, 122, 123]. However, the required energy to initiate the water splitting reaction should be at least 1.6 eV greater than the minimum energy of water splitting (1.23 eV), owing to the overpotentials from HER and even more from sluggish OER [124, 125, 126, 127, 128, 129, 130]. Additionally, only 43% of the sunlight or solar photons have energy larger than 1.6 eV [44]. Unlike water splitting, the oxidation of solid wastes is much easier because of the lower potential (∼0.12 eV), hence alleviating the overall energy demanding to couple with H_2_ evolution. Indeed, the photoreforming reaction of solid wastes and water into H_2_ is almost energy‐neutral [10, 48, 131, 132]. For example, the oxidation reaction of ethylene glycol and lactic acid with Gibbs free energy change (∆*G*) values of 9.2 and 27 kJ mol^−1^, respectively, is smaller than that of the OER of water splitting (∆*G* = 237 kJ mol^−1^) [48]. As a result, solid waste photoreforming requires much less energy than water splitting, which can enclose a larger range of the light spectrum, including visible and infrared bands.

**FIGURE 5:**
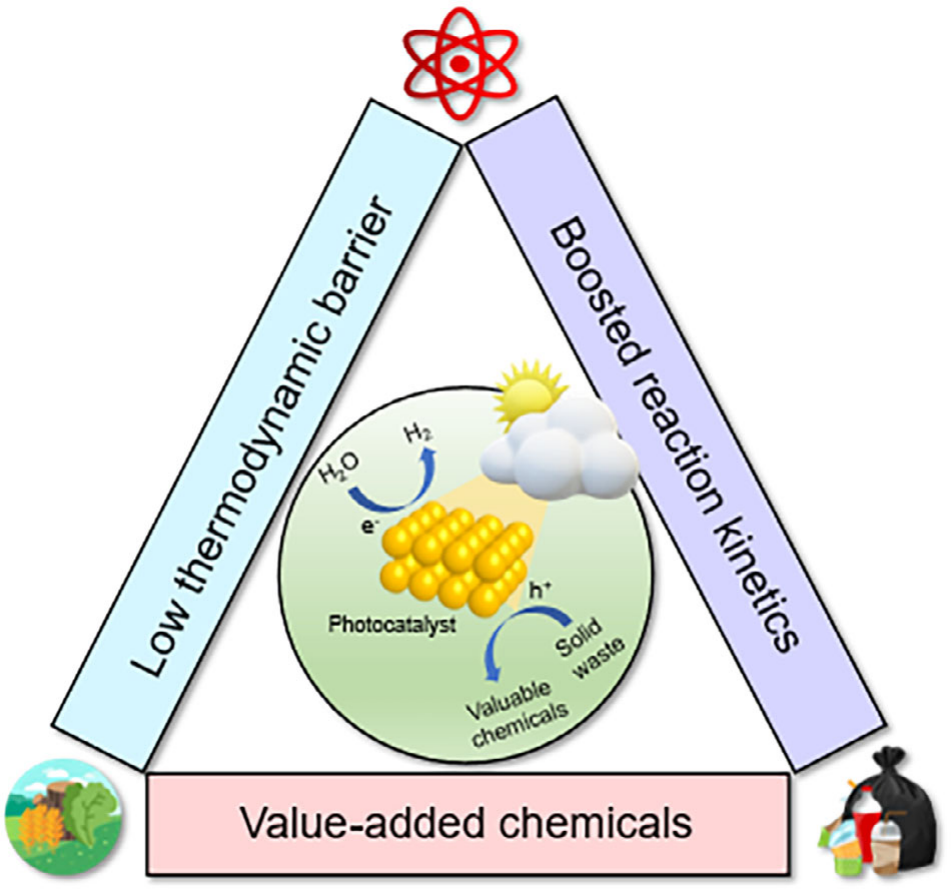
The advantages toward photoreforming of solid waste.

Generally speaking, the advantages of photoreforming of solid wastes can be summarized into three points in contrast to the photocatalytic water splitting reaction (Figure 5): (1) the oxidation reaction of solid wastes with less energy input can replace the OER with sluggish reaction kinetics to enhance the HER activity; (2) the replacement of solid waste oxidation for OER avoids the reverse reaction of O_2_ and H_2_; (3) solid wastes are used as chemical resources and further transformed to value‐added chemicals [110]. In addition, different from the common thermal‐assisted strategy toward catalytic upcycling of solid wastes under harsh conditions such as high temperature and/or high pressure, the photoreforming strategy can realize the upgrading of wastes under mild and environmentally benign conditions and, meanwhile, produce clean H_2_ fuel. Besides the aforementioned ethylene glycol and lactic acid, other solid wastes (e.g. fructose, polyethylene glycol, 5‐hydroxymethylfurfural) that possess high reproducibility and enthalpy have also been extensively utilized as feedstock to facilitate the HER [102, 133]. Consequently, the cooperative coupling of HER and solid waste oxidation leads to higher economic values in comparison with independent HER or solid waste oxidation [110].

## 3 Utilization of Photocatalysts in the Production of H_2_ and Value‐added Chemicals

The pioneer study of solid waste conversion to H_2_ through photocatalysis can be traced back to 1981. Sakata and Kawai realized the solid waste photoreforming by applying a platinized titania photocatalyst at room temperature, but their research was limited by the catalytic activity and low absorption efficiency of solar spectrum by TiO_2_ [134]. Owing to the different band gap structures in the semiconductors, considerable studies have been conducted in the development of a variety of photocatalysts to improve the solid waste photoreforming. For example, Reisner et al. recently utilized plastics and lignocellulose as feedstock to yield hydrogen with a CdS/CdO*_x_* photocatalyst under irradiation of visible light [59, 135]. Basically, photocatalysts can be mainly categorized into metal oxide photocatalysts, metal sulfide photocatalysts, metal‐free photocatalysts, and homogeneous photocatalysts. Diverse photocatalyst for biomass and plastic waste photoreforming have summarized in Tables 1 and 2.

**TABLE 1:** Summary of diverse photocatalysts for plastic waste photoreforming.

Photocatalyst	Plastic type	Synthesis method	Light source	Reaction time (h)	Product	Performance	Ref.
Ag_2_O/Fe‐MOF	PE PEG PET	Hydrothermal followed by post‐synthetic metal ion metathesis	300 W Xe lamp (AM 1.5 G)	2.5	H_2_ Acetic acid Ethanol Formic acid	H_2_ yield from PEG: 6.2 mmol g^−1^ h^−1^ H_2_ yield from PE: 1.70 mmol g^−1^ h^−1^ H_2_ yield from PET: 1.90 mmol g^−1^ h^−1^ Acetic acid yield from PEG: ∼58 mg l^−1^	[150]
Nb_2_O_5_	PE PP PVC	Hydrothermal followed by calcination	300 W Xe lamp (AM 1.5 G)	30	Acetic acid (CH_3_COOH)	PE‐to‐CH_3_COOH: 47.4 µg g_cat_^−1^ h^−1^ PP‐to‐CH_3_COOH: 40.6 µg g_cat_^−1^ h^−1^ PVC‐to‐CH_3_COOH: 39.5 µg g_cat_^−1^ h^−1^	[61]
nPpy@Fe_2_O_3_	PLA	Oxidative polymerization of pyrrole	Visible light household lamp (5 W cm^−2^)	168	H_2_ Lactic acid Formic acid Acetic acid	H_2_ yield from PLA: 78.6 mmol g^−1^ h^−1^	[227]
Co‐Ga_2_O_3_	PE PP PET	Hydrothermal	300 W Xe lamp (AM 1.5 G, 100 mW cm^−2^)	48	H_2_ CO_2_ CO	H_2_ yield from PE: 647.8 µmol g^−1^ h^−1^ H_2_ yield from PP: 603.5 µmol g^−1^ h^−1^ H_2_ yield from PET: 384.2 µmol g^−1^ h^−1^	[50]
Bi_4_V_2_O_11_‐O_V_	PET	Hydrothermal	300 W Xe lamp (AM 1.5 G, 150 mW cm^−2^)	5	CO Acetic acid Formic acid Glyoxylate Glyoxal	CO yield: 64.7 µmol g^−1^ h^−1^ Glyoxal yield: 6.9 mmol g_cat_^−1^ Glyoxylate yield: 3.2 mmol g_cat_^−1^	[228]
Pt/TiO_2_	PET	Rapid breakdown anodization‐photodeposition	300 W Xe lamp (320 nm ≤ λ ≤ 780 nm)	72	H_2_ Glyoxylate Glyoxal Lactate Acetate	H_2_ yield: 219.1 µmol g_cat_^−1^ h^−1^ Glyoxal yield: 0.039 mmol Glyoxylate yield: 0.045 mmol Lactate yield: 0.075 mmol Acetate yield: 0.069 mmol	[229]
BiVO_4_/MoO*_X_*	PET	Hydrothermal	300 W Xe lamp	5	H_2_ Formate Acetate Glycolate	Conversion of ethylene glycol: 11.2% Formate yield: 0.29 mmol g^−1^ h^−1^ Acetate yield: 0.19 mmol g^−1^ h^−1^ H_2_ yield: 1.96 mmol g^−1^ h^−1^	[230]
Ni_2_P‐Co_2_P/ZrO_2_/C nanofibers	PET	Integration of electrospinning, calcination, hydrothermal, and phosphorylation	500 W Hg lamp	24	H_2_ Formate Acetate Glycolate	H_2_ yield: 207.56 µmol g^−1^	[231]
Pt|TiO_2_	PE PP PVC	Photodeposition	Xe lamp	16	H_2_	H_2_ yield from PE: 103.21 µmol g^−1^ h^−1^ H_2_ yield from PP: 56.32 µmol g^−1^ h^−1^ H_2_ yield from PVC: 69.64 µmol g^−1^ h^−1^	[88]
MoS_2_/Cd_0.5_Zn_0.5_S	PET	Hydrothermal and calcination	300 W Xe lamp (AM 1.5 G)	5	H_2_ Formate Methanol Ethanol	H_2_ yield: 15.90 mmol g^−1^ h^−1^	[58]
MXene/Zn_0.6_Cd_0.4_S	PET	Etching process and Solvothermal	300 W Xe lamp (λ > 420 nm)	4	H_2_ Glycolate Acetate Methanol	H_2_ yield: 14.17 mmol g^−1^ h^−1^	[166]
CdS/CdO*_x_*	PET PLA PUR	Wet chemical approach	Simulated solar light (100 mW cm^−2^, AM 1.5 G)	4	H_2_ Formate Glycolate Ethanol Acetate Lactate Pyruvate	PET conversion: 16.6 ± 1.0% PLA conversion: 38.8 ± 4.0% PUR conversion: 22.5 ± 3.4% H_2_ yield from PET: 3.42 ± 0.87 mmol g^−1^ h^−1^ H_2_ yield from PLA: 64.3 ± 14.7 mmol g^−1^ h^−1^ H_2_ yield from PUR: 0.85 ± 0.28 mmol g^−1^ h^−1^ AQY: 2.17 ± 0.38%	[59]
CdS/MoS_2_	PE PET PLA	Hydrothermal and calcination	300 W Xe lamp (AM 1.5 G)	25	H_2_ Formate Acetate Glycolate Methane Ethane Propane N‐pentane	H_2_ yield from PE: 1.13 ± 0.06 mmol g^−1^ h^−1^ H_2_ yield from PET: 3.9 ± 0.07 mmol g^−1^ h^−1^ H_2_ yield from PLA: 6.2 ± 0.23 mmol g^−1^ h^−1^	[167]
CdS/CdO*_x_*/SiC	PE PS Lignin	Wet chemical approach	300 W Xe lamp	3	H_2_	H_2_ yield from PE: 25.0 µmol g^−1^ h^−1^ H_2_ yield from PS: 19.4 µmol g^−1^ h^−1^ H_2_ yield from Lignin: 71.5 µmol g^−1^ h^−1^ AQY: 2.0%	[232]
Pd‐CdS	PLA	Wet chemical approach‐chemical reduction	300 W Xe lamp (λ > 400 nm, 1.1 W cm^−2^)	12	H_2_ Pyruvic acid	H_2_ yield: 1121 µmol Pyruvic acid yield: 958.7 µmol Selectivity toward pyruvic acid: 86.8%	[57]
Mesoporous ZnIn_2_S_4_	PET PLA	Wet chemical approach	Solar light simulator (AM 1.5G, 150 mW cm^−2^)	20	H_2_ Pyruvic acid Acetic acid Glycolic acid	H_2_ yield from PLA: 412.1 µmol g^−1^ H_2_ yield from PET: 472.3 µmol g^−1^	[233]
Ni_2_P/ ZnIn_2_S_4_	PLA PET PTT PBT	Hydrothermal	White LED light (λ > 420 nm, 4 × 25 W)	5	H_2_ Pyruvic acid Formate Acetate Glycolate Malonate Succinic acid	H_2_ yield from PLA: 781.3 µmol g^−1^ h^−1^ H_2_ yield from PET: 686.1 µmol g^−1^ h^−1^ H_2_ yield from PTT: 519.4 µmol g^−1^ h^−1^ H_2_ yield from PBT: 330.0 µmol g^−1^ h^−1^ Pyruvic acid yield: 745.9 µmol g^−1^ h^−1^	[234]
g‐C_3_N_4_/CdS/NiS	PLA	Thermal polymerization‐hydrothermal	300 W Xe lamp (visible light)	5	H_2_ Formate Acetate Pyruvate	H_2_ yield from PLA in seawater: 30.44 mmol g^−1^ h^−1^ H_2_ yield from PLA in pure water: 25.79 mmol g^−1^ h^−1^	[235]
d‐NiPS_3_/CdS	PET PLA	Combination of exfoliation‐plasma method and hydrothermal	300 W Xe lamp (λ > 400 nm)	1	H_2_ Formate Acetate Pyruvate Glycolate	H_2_ yield from PLA: 39.76 mmol g_cat_^−1^ h^−1^ H_2_ yield from PET: 31.38 mmol g_cat_^−1^ h^−1^ Acetate yield from PLA: 13.6 µmol^*^ Pyruvate yield from PLA: 64.5 µmol^*^ Formate yield from PET: 23.6 µmol^*^ Acetate yield from PET: 13.8 µmol^*^ Glycolate yield from PET: 25 µmol^*^ *: 9 h reaction	[52]
CPDs‐CN	PET PLA	Hydrothermal	300 W Xe lamp (λ > 420 nm)	−	H_2_ Acetic acid Formic acid Glycollic acid Ethanol Acetaldehyde Glycolaldehyde	H_2_ yield from PET: 515 ± 168 µmol g^−1^ h^−1^ H_2_ yield from PLA: 247 ± 38 µmol g^−1^ h^−1^ AQY: 2.55% (420 nm)	[236]
CN‐CNTs‐NiMo	PET	Thermal condensation‐hydrothermal‐calcination	500 W Xe lamp (stimulated solar light, 95 mW cm^−2^)	4	H_2_ Glyoxal Glycolate	H_2_ yield: 90 µmol g^−1^ h^−1^ AQY: 0.56% (420 ± 5 nm)	[194]
g‐C_3_N_4_	PS	Thermal condensation	300 W Xe lamp (400‐800 nm filter)	24	Benzoic acid Acetophenone Benzaldehyde CO_2_	PS conversion: 96 ± 6% Product selectivity: 60 ± 4%	[65]
Ni*_x_* Co_1−_ *_x_* P/rGO/g‐ C_3_N_4_	PLA	Thermal condensation‐hydrothermal‐calcination	300 W Xe lamp, 150°C	50	H_2_	H_2_ yield: 576.7 µmol g^−1^ h^−1^ AQY: 1.7% (420 nm)	[199]
^NCN^CN*_x_* |Pt	PE	Thermal polymerization	Solar light simulator (100 mW cm^−2^, AM 1.5 G)	72	Ethane Propane CO_2_	PE‐to‐ethane yield: 7.2 µmol g_cat_^−1^ h^−1^ PE‐to‐ethylene yield: 1.3 µmol g_cat_^−1^ h^−1^ PE‐to‐H_2_ yield: 137 µmol g_cat_^−1^ h^−1^	[90]
^H₂N^CN*_x_* |Ni_2_P	PET PLA	Thermal polymerization‐phosphorylation	Solar light simulator (100 mW cm^−2^, AM 1.5 G)	20	H_2_ Formate Acetate Glycolate Glyoxal	H_2_ yield from PET: 141 ± 16 µmol g_sub_^−1^ H_2_ yield from PLA: 427 ± 21 µmol g_sub_^−1^ PET AQY: 0.035 ± 0.005% (430 nm) PLA AQY: 0.101 ± 0.018% (430 nm)	[48]
VPOM/g‐C_3_N_4_	PE PP PVC PEG	Self‐assembly strategy	300 W Xe lamp (λ > 420 nm)	36	Formic acid	Formic acid yield from PE: 24.66 µmol g^−1^ h^−1^ Formic acid from PP: 26.68 µmol g^−1^ h^−1^ Formic acid from PVC: 29.85 µmol g^−1^ h^−1^ Formic acid from PEG: 208.65 µmol g^−1^ h^−1^	[206]
g‐C_3_N_4_/CuFeO_2_	Polyester microfiber	Thermal treatment	Solar light simulator (100 mW cm^−2^, AM 1.5 G)	48	H_2_	H_2_ yield in seawater: ≥3000 µmol g_cat_^−1^	[237]
Polymeric g‐C_3_N_4_/WO_3_	PLA	Annealing	300 W Xe lamp (λ > 420 nm)	4	H_2_ Formate Acetate	H_2_ yield: 402.9 µmol g^−1^ h^−1^ Formate yield: ∼3.0 µmol Acetate yield: ∼2.0 µmol	[238]
Carbon nitride porous microtubes	PET	Calcination	300 W Xe arc lamp (λ > 420 nm)	4	H_2_ Formate Acetate Glycolate Glyoxal	H_2_ yield: 39.65 µmol g^−1^ Formate yield: 10340 nmol Acetate yield: 860 nmol Glyoxal yield: 7320 nmol	[239]
Ultrathin porous C_3_N_4_|Pt nanoclusters	PET	Thermal polymerization‐photodeposition	300 W Xe lamp	3	H_2_ Formate Acetate Glycolate Glyoxal	H_2_ yield: 11.69 mmol g_cat_^−1^ h^−1^ AQY: 23.9% (365 nm)	[208]
g‐C_3_N_4_/Pt	PET	Thermal polymerization and photodeposition	100 W Xe lamp (100 mW cm^−2^, AM 1.5 G)	12	H_2_ Formate Acetate Glycolate Glyoxal	H_2_ yield: 7.33 ± 0.021 mmol g_cat_^−1^ h^−1^	[209]
CN*_x_* |Pt on hollow glass microspheres	PET Cellulose	Calcination and chemical reduction	Solar light simulator (100 mW cm^−2^, AM 1.5 G)	20	H_2_	Aerial activity in PET: 31.1 ± 12.1% Aerial activity in cellulose: 67.5 ± 11.4%	[240]
Vanadium (V) complexes	PEG 400 PCL‐PEG‐PCL PE‐PEG PE‐monoalcohol	Schlenk technique	White LED	> 48	Formic acid Methyl formate	PEG 400 conversion: > 95% PCL‐PEG‐PCL conversion: > 95% PE‐PEG conversion: > 95% PE‐monoalcohol conversion: > 95% PEG 400‐to‐methyl formate: 75 ± 4% PCL‐PEG‐PCL‐to‐formic acid: 70 ± 4% PE‐PEG‐to‐formic acid: 6 ± 1% PE‐monoalcohol‐to‐formic acid: 5 ± 2%	[241]
FeCl_2_	PS	−	LED (400 nm)	66	Benzoic acid	PS conversion: 100% Benzoic acid yield: 63%	[242]
*N*‐bromosuccinimide	PS	−	Kessil light (390 nm)	16	Benzoic acid Benzaldehyde Acetophenone Benzoyl bromide	Benzoic acid yield: 73%	[226]
pTsOH • H_2_O	PS	−	Violet blue light (405 nm)	15	Formic acid Benzoic acid Benzophenone	Formic acid yield: 67% Benzoic acid yield: 50% Benzophenone yield: 2%	[214]
FeCl_3_	PS	−	White LED lamp, O_2_	20	Benzoic acid Benzaldehyde Benzoyl chloride Acetophenone	Total yield of benzoyl products: 22.8 mol %	[222]
Binuclear zinc complex/g‐C_3_N_4_/Pt	PET	Calcination and chemical reduction	Xe lamp (λ > 420 nm)	4	H_2_ Formic acid	PET‐to‐monomers: >90% H_2_ yield: 400 µmol Formic acid yield: 62 µmol	[56]
Ag‐PEDOT‐COF	PET PLA	−	48 W LED household lamp (20 mW cm^−2^)	24	H_2_ Glycolate Methanol Acetate Ethanol	H_2_ yield from PET: 113.4 mmol g_cat_^−1^ h^−1^ H_2_ yield from PLA: 95.56 mmol g_cat_^−1^ h^−1^	[243]
Re‐Ru‐Bipyridine‐silica nanotubes (Re‐Ru‐Bpy‐NT)	PET	Hydrothermal and wet chemical approach	300 W Xe lamp (λ > 420 nm)	12	CO Formic acid Acetic acid Glycolic acid	TON for CO: 8.8 Selectivity toward CO: 95% Selectivity toward formic acid: 65% Selectivity toward acetic acid: 27% Selectivity toward glycolic acid: 8%	[111]
Fluorenone	PS	−	Blue LED (*λ*_max_ ∼ 450 nm)	16	Benzoic acid Ethyl benzoate Acetophenone Benzaldehyde Phenylglyoxylic acid	Benzoic acid yield: 30 ± 2%	[216]

**TABLE 2:** Summary of diverse photocatalysts for biomass photoreforming.

Photocatalyst	Biomass type	Synthesis method	Light source	Reaction time (h)	Product	Performance	Ref.
Ni*_x_* S*_y_*‐TiO_2_	Cellulose	Refluxing with N_2_ bubbling	500 W Xe lamp (400 mW cm^−2^)	3	H_2_ Formic acid	HER rate: 3.02 mmol g^−1^ h^−1^	[244]
MoS_2_/TiO_2_	Lignocellulose	Hydrothermal	300 W Xe lamp	6	H_2_ Glucuronic Galaturonic acid Glucose N‐acetyl‐galactose arabinose	HER rate: 21.4 µmol g^−1^ h^−1^ AQY: 1.45% (380 nm)	[245]
TiO_2_/NiO*_x_* @C_g_	Cellulose Glucose Glycol	Hydrothermal	500 W Xe lamp	5	H_2_ CO CO_2_ CH_4_	HER rate: 270 µmol g^−1^ h^−1^	[246]
TiO_2_/C	Lignin	Sol‐microwave	Mercury lamp (400 W, *λ*_max_ = 365 nm)	5	Butanedinitrile Benzoic acid Vanilline Apocynin BHT Isovanillic acid Propiovanillone Coniferyl aldehyde	Lignin conversion: 40.28% (UV irradiation) Lignin conversion: 15.73% (Visible light irradiation)	[247]
Pt/m‐TiO_2_	Glucose	Impregnation	UV‐A lamp (2 × 8 W bulbs, *λ*_max_ = 365 nm)	3	H_2_ Arabinose Formic acid	Selectivity toward arabinose: 45–52% Selectivity toward formic acid: 37–43%	[248]
TiO_2_‐NiO	Lignin	Hydrothermal	UV–vis light (320–780 nm)	5	H_2_ Methane Palmitic acid Stearic acid Butanedioic acid	HER rate: 23.53 mmol g^−1^ h^−1^ AQY: 6.46% (365 nm)	[148]
Ta‐doped CeO_2_	Cellulose Hemicellulose Lignin	Hydrothermal	Blue LED light (*λ* = 452 ± 10 nm)	−	Formic acid Formaldehyde	Selectivity toward C_1_ product of poplar: 74% Selectivity toward C_1_ product of wheat straw: 65%	[249]
h‐ZnSe/Pt@TiO_2_	Cellulose	Wet chemical approach and annealing	300 W Xe lamp	300	H_2_ Formic acid	H_2_ yield: 380 mmol g^−1^ Formic acid yield: 372 µmol g^−1^ h^−1^	[250]
CdS QDs	Lignin	Hot‐injection	300 W Xe lamp (UV cut off filter)	8	Aromatic monomers	Aromatic monomer yield: 27 wt%	[49]
CdS/CdO*_x_*	Lignin Hemicellulose α‐cellulose Cellulose	Hot‐injection	Newport Oriel 100 mW cm^−2^ (AM 1.5 G)	24	H_2_ Formate CO_2_	Lignin HER rate: 0.026 mmol g^−1^ h^−1^ Hemicellulose HER rate: 2 mmol g^−1^ h^−1^ α‐cellulose HER rate: 2.5 mmol g^−1^ h^−1^ AQY: 1.2 ± 0.4%	[135]
Ni_2_P/CdS	Cellulose Hemicellulose Lignin	Solvothermal and annealing	300 W Xe lamp	2	H_2_	H_2_ yield from cellulose: 534.3 µmol g^−1^ h^−1^ H_2_ yield from hemicellulose: 382.2 µmol g^−1^ h^−1^ H_2_ yield from lignin: 322.8 µmol g^−1^ h^−1^	[251]
CdS/TiO_2_/Biochar	Glucose	Hydrothermal	300 W Xe lamp		H_2_ Formic acid Acetic acid	H_2_ yield in NaOH solution: ∼12.77 mmol g^−1^ h^−1^ H_2_ yield in Na_2_CO_3_ solution: ∼10.29 mmol g^−1^ h^−1^ Selectivity toward acetic acid: 63.94% Selectivity toward formic acid: 60.29%	[252]
CdS/MoS_2_	FFA PLA PET	Hydrothermal	300 W Xe lamp (*λ* = 420 nm)	8	H_2_	FFA HER rate: 5.285 mmol g^−1^ h^−1^ PET HER rate: 60.775 mmol g^−1^ h^−1^ PLA HER rate: 379.5 mmol g^−1^ h^−1^ AQY: 16.64%	[253]
Ti_3_C_2_T*_x_*/CdS	FFA	Refluxing with N_2_ atmosphere	300 W Xe lamp (*λ* > 420 nm)	4	H_2_ Furfural	H_2_ yield: 773 µmol g^−1^ Furfural yield: 777 µmol g^−1^ FFA conversion: ∼ 100%	[254]
LaVO_4_/g‐C_3_N_4_	FFA	Hydrothermal with thermal treatment	300 W Xe lamp	5	H_2_ Furfural	FFA HER: 1.44 mmol g^−1^ h^−1^ AQY: 2.03% (500 nm)	[255]
UCN‐NA	Lignocellulose	Thermal pyrolysis	White LED (5 W)	5	H_2_	H_2_ yield: 136.9 µmol	[256]
TiO_2_‐Pt	Lignocellulose	Hydrothermal	300 W Xe lamp	4	H_2_ Lactic acid Arabinose Glucose Mannose	HER rate: 275 µmol g^−1^ h^−1^ AQY: 1.89% (380 nm) TOC: 195.7 mg L^−1^	[149]
Ni/CdS	FFA HMF	Microwave treatment followed by chemical reduction	Blue LED (450 nm, 8 W)	8	H_2_ Furfural Furoic acid DFF FDCA	FFA HER rate: 4.5 mL HMF HER rate: 0.5 mL FFA‐to‐furfural: ∼100%	[63]
g‐C_3_N_4_	HMF	Thermal polymerization	370–467 nm	12	DFF	DFF yield: 73% HMF conversion: 75% AQY: 11.7% (390 nm)	[257]
Pt‐C_3_N_4_	Cellulose Hemicellulose Lignin	Supramolecular self‐assembly strategy	LED light (427 nm)	−	H_2_	HER; 3.39 mmol g^−1^ h^−1^	[51]
Cu, In‐doped ZnS	α‐cellulose	Hydrothermal	Solar simulator (AM 1.5 G)	3	H_2_ Monosaccharides HMF Organic acids	HER rate: 74.8 µmol g^−1^ h^−1^ AQY: 27.94% (360 nm) AQY: ∼ 0% (420 nm)	[174]
CdS/SiC	Lignin α‐cellulose	Sol‐gel	300 W Xe lamp (*λ* > 420 nm)	−	H_2_	α‐cellulose HER rate: 321.7 µmol g^−1^ h^−1^ Lignin HER rate: 11.0 µmol g^−1^ h^−1^ AQY: 19.6% (380 nm) AQY: 2 % (420 nm)	[258]
S, N‐doped GODs	Cellulose	Modified Hummer's method	Simulated solar light (AM 1.5 G, 100 mW cm^−2^)	24	H_2_ Formate	HER rate: ∼ 100 µmol h^−1^ (after 6 days of irradiation)	[64]
^NCN^CN*_x_* |Pt	α‐cellulose	Thermal polymerization	Simulated solar light (AM 1.5 G, 100 mW cm^−2^)	144	H_2_ Formate Glucose Carboxylate group	HER rate: 39.5 ± 1.1 µmol Conversion: 22%	[47]
α‐cel‐carbon Dots/NiP	Cellulose	Thermal treatment	Simulated solar light (AM 1.5 G, 100 mW cm^−2^)	24	H_2_	HER rate: 13405 µmol g^−1^ h^−1^ IQE: 11.4% (360 nm)	[207]
Carbon nitride, CN*_x_*	Xylose	Thermal polymerization	Visible light source	2.5	Lactic acid	Lactic acid yield: ∼75% Xylose conversion: ∼100%	[259]
Au‐Pt/g‐C_3_N_4_	Cellulose	Thermal polymerization and photodeposition	300 W Xe lamp (150 mW cm^−2^, 350–800 nm)	4	H_2_	H_2_ yield: 1068.8 µmol g^−1^ h^−1^ AQY: 1.74% (420 nm)	[260]
PCN/KS‐OH	Cellubiose	Thermal polymerization	300 W Xe lamp (*λ* > 400 nm)	6	H_2_ Glucose Gluconic acid CO	H_2_ yield: 56 µmol g^−1^ Cellobiose‐to‐Glucose conversion and yield: >90% and ∼1.8 g L^−1^ Cellobiose‐to‐Gluconic conversion and yield: >80% and ∼55%	[261]
Pt*_x_*‐C_3_N_4_	Gluscose	Supramolecular assembly strategy	40 W blue LED (*λ* = 427 nm)	4	H_2_ Lactic acid	H_2_ yield: 3.39 mmol g^−1^ h^−1^ Glucose conversion: 100% Selectivity toward lactic acid: 86%	[51]
C‐doped PCN	Glucose	Calcination	300 W Xe lamp (0.99 W cm^−2^)	3	Glycerol	Glucose conversion: >95% Glycerol yield: ∼50% Selectivity toward glycerol: >50%	[262]
O_2_‐doped CN	Glucose	Solvothermal	300 W Xe lamp (AM 1.5 G)	6	Arabinose Gluconic acid Formic acid	Glucose conversion: ∼10% Selectivity toward arabinose: ∼30% Selectivity toward gluconic acid: ∼40% Selectivity toward formic acid: ∼10%	[263]
Fe‐SA/PCN homojunction	Fructose	Calcination	300 W Xe lamp	2	CO Lactic acid	CO yield: 92.33 mmol g^−1^ Lactic acid yield: 136.21 mg AQY: 44.2% (420 nm)	[264]
g‐C_3_N_4_‐TiO_2_@montmorillonite	Oleic acid	Calcination	300 W Xe lamp (*λ* > 400 nm)	2	Biodiesel	Biodiesel yield: 97.6%	[265]
Si flakes/Ni‐doped graphene quantum dots	Kraft lignin	Hydrothermal‐magnesiothermic reduction	450 W Xe lamp (AM 1.5 G)	6	H_2_ Guaiacol Vanillin Acetovanillone	H_2_ yield: 14.27 mmol g^−1^ h^−1^ Vanillin yield: 150 mg	[53]
Urea‐incorporated (TBUPP)‐Cu MOF	HMF	Wet chemical approach	LED light (1 W, *λ* = 430 nm)	24	2,5‐furandicarboxylic acid (FDCA)	Selectivity toward FDCA: ∼90% HMF conversion: >95%	[55]
TEMPO/Zr‐RuCP^6^‐Zr‐RuP^6^@Pt‐TiO_2_	Glycerol Cellulose	−	Blue LED light (*λ* = 460 nm ± 15 nm, 80 mW)	24	H_2_	H_2_ yield from glycerol: 2.67 mmol g_cat_^−1^ h^−1^ H_2_ yield from cellulose: 1.59 mmol g_cat_^−1^ h^−1^	[54]

### 3.1 Metal Oxide

Over the past few decades, several metal oxide photocatalysts, such as TiO_2_, Nb_2_O_5_, Ga_2_O_3_, Ag_2_O etc., have been used in the photocatalysis due to their following excellent properties: (i) different crystal structures of metal oxide can be obtained through various synthetic methods, which are facile for modification strategies (e.g. composite construction, heteroatom doping, heterojunction fabrication, morphology control); (ii) the metal oxide nanoparticles always have high specific surface area, abundant active sites, and numerous exposed atoms, leading to the reduction of the distance of charge movement, thus facilitating photocatalysis process [136, 137]. Up to now, TiO_2_ is one of the most widely used semiconductors in the photocatalytic conversion of solid waste owing to its low toxicity, abundance, cost effectiveness, high stability, and high photooxidation ability. [138, 139, 140, 141, 142, 143, 144, 145, 146, 147] However, TiO_2_ possesses a wide band gap (3.2 eV), which would result in a poor photocatalytic activity under visible light. Moreover, the high charge recombination rate of TiO_2_ can decrease the quantum and photocatalytic efficiency of TiO_2_ in the redox reaction [138]. To address these issues, the catalyst modifications, such as cocatalyst doping with metals (e.g. Pt, Ni, Ag and other metals) and non‐metals (e.g. S, N, and C) and formation of heterojunction composites with a narrow band gap semiconductor, can significantly enhance the absorption of visible light and separation of charge carrier [44, 138].

Recently, Zhao et al. succeeded to photoreform the lignin into H_2_ and organic compounds using a n‐p heterojunction of TiO_2_@NiO photocatalyst under UV and visible light [148]. The p‐type NiO is discovered to be an effective semiconductor for lignin conversion, indicating that the photoinduced holes accumulated in the VB of NiO are the key factor for lignin valorization. Also, the type II heterojunction can provide a strong internal electric field, which help promotes the efficient electron–hole pairs separation and rapid charge transfer for the redox reaction. Thus, with the optimal Ni content, this core–shell TiO_2_‐NiO photocatalyst exhibits significant improvement in the hydrogen evolution reaction compared with the TiO_2_ and NiO sample. Interestingly, methane was detected as another gaseous product, whereby the lignin was fully decomposed into CO_2_ and then the CO_2_ was further photoreduced into CH_4_. In addition, the aqueous products from lignin including palmitic acid (35%), stearic acid (25%), and butanedioic acid (7%) were observed using gas chromatography‐mass spectroscopy (GC‐MS). A similar result was also obtained by Cheng et al. who valorized the lignocellulose over an ultrathin anatase TiO_2_/Pt nanosheets to produce organic acids and carbohydrates [149].

**FIGURE 6:**
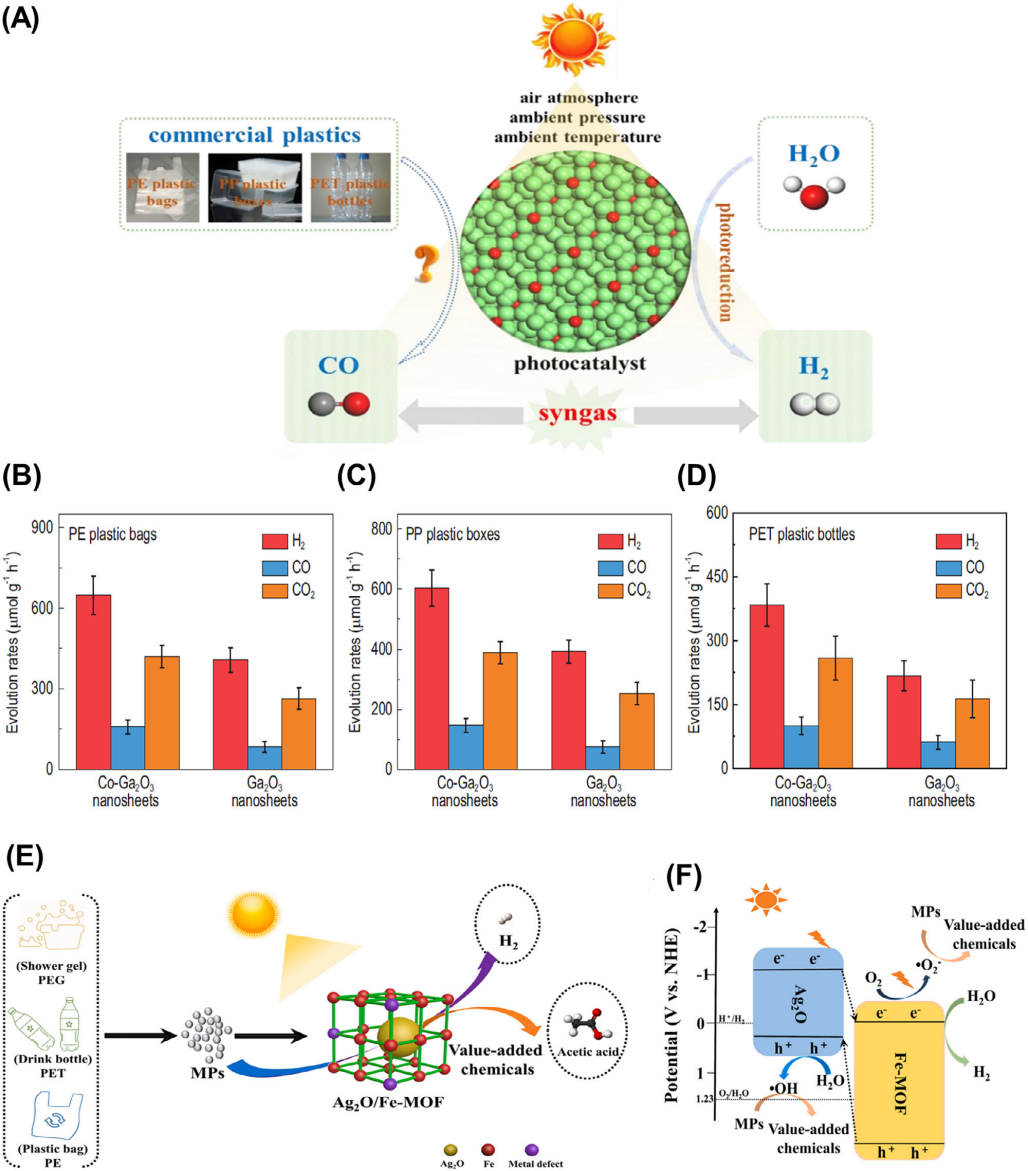
(A) Scheme of the photoconversion process of plastics into syngas under mild conditions. The evolution rates of H_2_, CO, and CO_2_ from (B) PE plastics bags (C) PP plastic boxes (D) PET plastics bottles for Co‐Ga_2_O_3_ and Ga_2_O_3_ nanosheets [50]. Reproduced under the terms of the Creative Commons CC BY license [50]. Copyright 2022, Oxford University Press. (E) Scheme of the heterostructure of Ag_2_O/Fe‐MOF and its application in upcycling microplastics. (F) Reaction mechanism on Ag_2_O/Fe‐MOF photocatalyst for microplastic conversion [150]. Reproduced with the permission of Qin et al. [150] Copyright 2022, Elsevier.

Another notable example was performed by Xu et al., who reported the photosynthesis of PE plastic bags, PP plastic boxes and PET plastics bottles into syngas by employing Co‐Ga_2_O_3_ nanosheets as photocatalysts under mild conditions, as shown in Figure 6A [50]. The introduction of the Co atom into the pristine Ga_2_O_3_ nanosheets via doping could restrain the recombination rate of photoinduced electron–hole pairs and extend the visible light absorption range. The position of CB and VB for Co doped Ga_2_O_3_ nanosheets was located at −1.4 and 2.5 V vs. NHE (normal hydrogen electrode) at pH 7, respectively. Meanwhile, the edge of CB and VB for Ga_2_O_3_ nanosheets was located at −1.46 and 3.19 V vs. NHE at pH 7 [50]. Taking PE as an example, under the simulated sunlight irradiation, the Co‐Ga_2_O_3_ photocatalyst was able to convert the pulverized PE into H_2_, CO_2_, and CO with an evolution rate of 647.8, 419.3, and 158.3 µmol g^−1^ h^−1^, respectively, which was around 1.6, 1.6, and 1.9 times greater than that of the pristine Ga_2_O_3_ nanosheets (Figure 6B–D) [50]. However, the syngas evolution process has revealed that the photogenerated holes were underutilized owing to the production of CO_2_ during the photoreforming process.

To fully utilize the value of solid wastes, Qin and coworkers successfully prepared an Ag‐based photocatalyst by encapsulating the Ag_2_O nanoparticles (p‐type semiconductor) within the porous Fe‐MOF (n‐type semiconductor) to convert PE, polyethylene glycol (PEG) and polyethylene terephthalate (PET) into small hydrocarbon molecules, and meanwhile generate H_2_ (Figure 6E) [150]. The photoreforming of PE, PEG, and PET over Ag_2_O/Fe‐MOF generated H_2_ with respective formation rates of 1.70, 2.48, and 1.90 mmol g^−1^ h^−1^, which were higher than the previous report using Co‐doped Ga_2_O_3_ nanosheets. In this study, the pretreatment of plastics in a strong alkaline solution also plays a vital role in facilitating the hydrolysis of PE, PEG, and PET into their monomers, which can be readily photoconverted [44]. Different from producing CO and CO_2_ by Co‐Ga_2_O_3_, this Ag_2_O/Fe‐MOF photocatalyst was also capable of oxidizing the primary monomers of plastics into value‐added chemicals, including ethanol, formic acid, and acetate via an indirect pathway. Owing to its transfer pathway based on type II heterojunction, the photoexcited electrons in the CB of Ag_2_O would be transferred to the CB of Fe‐MOF and involved in the generation of **·**O^−^_2_ and H_2_, while the photoinduced holes in the VB of Fe‐MOF transferred to the VB of Ag_2_O to engage in generating hydroxyl radicals (Figure 6F) [150]. Thereby, the plastics molecules can be effectively broken down into smaller organic molecules by the produced radicals [151, 152].

Although Ag/Fe‐MOF photocatalyst could yield H_2_ and high‐value chemicals simultaneously, the generation of organic products was nonselective in the previous report. As such, Jiao et al. realized a two‐step photoconversion process, comprising successive photoinduced C─C bond cleavage and coupling pathway, to selectively convert the plastic wastes into C_2_ fuel over a niobium‐based photocatalyst without applying a sacrificial agent [61]. Of note, the Nb_2_O_5_ atomic layers were used in the two‐step process as the position of VB and CB of Nb_2_O_5_ was located at 2.5 and −0.9 V vs. NHE at pH 7, which were adequate for the plastics oxidation [153]. Particularly, the whole photosynthesis reaction can be separated into two sequential processes. The plastics firstly would be decomposed into CO_2_ via C─C bond cleavage with the aid of oxygen and hydroxyl radicals. Then, the produced CO_2_ was reduced to acetate through C─C bond coupling of hydrocarboxyl radicals. Under simulated natural environment conditions, a variety of plastics including PE, PP, and polyvinyl chloride (PVC) were photoconverted into acetic acid with formation rates of 47.4, 40.6, 39.5 µg g_cat_^−1^ h^−1^, respectively. Hence, this two‐step photosynthesis reaction of selectively converting plastics into chemicals can be a beneficial paradigm in leading the design of photocatalytic conversion of solid wastes under mild conditions. Shortly, the metal oxide photocatalysts have large applicability due to their fascinating features such as suitable band gaps, reusability, and chemical and thermal stability. Both photocatalytic activity and stability also can be improved by various modification strategies.

### 3.2 Metal Sulfide

Regardless of single‐metal sulfide (e.g. ZnS, CdS, MoS_2_, Bi_2_S_3_, and Ag_2_S) [154, 155] and bimetallic sulfides (e.g. CdZnS, CdInS, ZnInS, NiCo_2_S_4_, and CuCo_2_S_4_) [58, 156, 157], metal sulfides often possess wide solar light harvesting ranges and great photocatalytic activities. In fact, sulfur can effectively decrease the width of the band gap, leading to an improvement in the visible light absorption capabilities of the metals [158]. Particularly, the heterojunction construction can be an impressive strategy to enhance the photocatalytic performance of metal sulfides [159]. To date, CdS have been extensively applied in the study of water splitting and other processes due to its wide band gap of 2.4 eV that enables the absorption of visible light [160]. Also, its CB (−0.5 V vs. NHE) and VB (1.9 V vs. NHE) position favors the photoreforming reactions. Unlike other photocatalysts, CdS has the ability to reduce water to H_2_ without the presence of a co‐catalyst [160, 161]. In this regard, Reisner et al. realized the valorization of PET, polylactic acid (PLA), and polyurethane (PUR) employing low‐cost CdS/CdO*_x_* quantum dots at room temperature and pressure [59]. The plastics were impregnated in the alkaline aqueous solution (10 m NaOH) for the hydrolysis process to enhance the photoreforming activity. In this work, H_2_ and several organic products, such as formic acid, ethanol, acetic acid, pyruvate, and lactate, were successfully detected using GC and ^1^H NMR.

**FIGURE 7:**
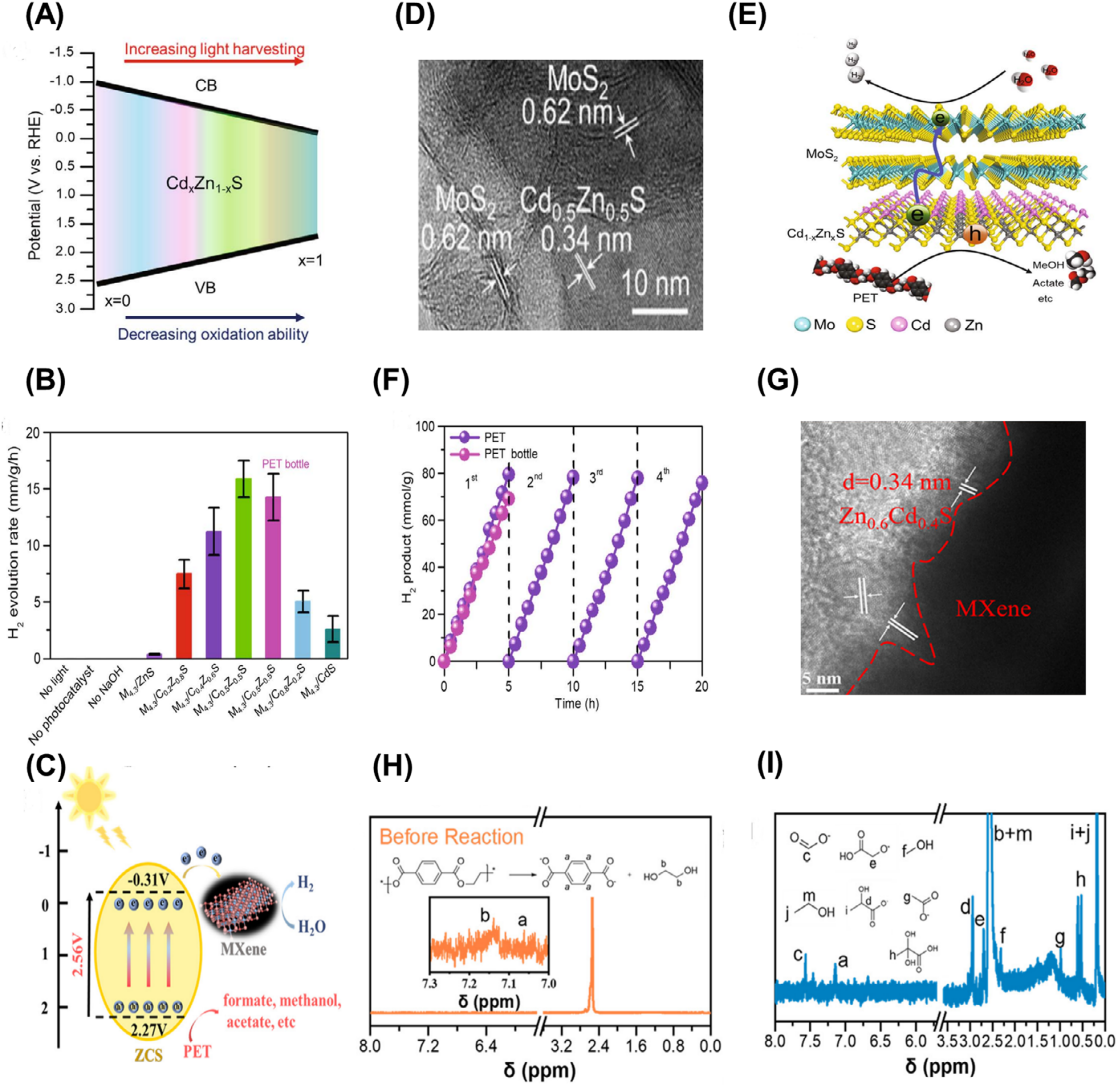
(A) Band structure alignments of Cd*_x_* Zn_1−_ *_x_* S. (B) HR‐TEM image of MoS_2_/Cd*_x_* Zn_1−_ *_x_* S composites [58]. Reproduced with the permission of Li et al. [58] Copyright 2021, Wiley‐VCH. (C) Scheme of mechanism for PET degradation coupled with H_2_ production over MXene/Cd*_x_* Zn_1−_ *_x_* S [166]. Reproduced with the permission of Cao et al. [166] Copyright 2021, Elsevier. (D) HRTEM image of MoS_2_/Cd*_x_* Zn_1−_ *_x_* S. (E) Scheme of charge transfer and H_2_ evolution reaction for MoS_2_/Cd*_x_* Zn_1−_ *_x_* S. (F) Photocatalytic H_2_ generation cyclic test of MoS_2_/Cd*_x_* Zn_1−_ *_x_* S. (G) HRTEM image of MXene/Cd*_x_* Zn_1−_ *_x_* S. ^1^H NMR spectra of MXene/Cd*_x_* Zn_1−_ *_x_* S (H) before and (I) after reaction [166]. Reproduced with the permission of Cao et al. [166] Copyright 2021, Elsevier.

Nonetheless, the redox ability and stability of metal sulfides were lower than that of metal oxides, which constrain their applications. For instance, the photogenerated holes by CdS have poorer oxidation capability to oxidize the solid wastes with respect to the thermodynamic viewpoint, wherein the substrates cannot play the same role as hole scavengers to consume the holes. The shallow VB position of CdS is claimed as a factor that restricts the rate of reaction owing to the weak oxidation ability [162]. To this end, the position of VB should be deepened for efficient solid waste oxidation and HER. Thence, Zn can be introduced into CdS to form a bimetallic sulfide, Cd*_x_* Zn_1−_ *_x_* S (0 ≤ *x* ≤ 1), which is a promising photocatalyst for enhancing the above‐mentioned redox reaction [163, 164]. Its band gap structure can be readily modified with the changes in the band gap and band edges by regulating the ratio of Cd/Zn (Figure 7A) [165].

Very lately, MoS_2_ and MXene were utilized as the cocatalyst to ameliorate Cd*_x_* Zn_1−_ *_x_* S for improving plastic photoreforming [58, 166]. The closely packed interfaces between the bimetallic semiconductor and cocatalysts were observed using HRTEM (Figure 7B,C), which were accountable for highly efficacious electron transfer in the photoreforming process. As presented in Figure 7D,E, the photocatalytic mechanisms basically involve three steps: (1) the photoexcited charged carriers are generated under the solar irradiation; (2) the photogenerated electrons would transfer to the surface of MXene or MoS_2_; (3) meantime, the photoinduced holes would convert PET into high‐value products including methanol, ethanol, formic acid, and acetate. The photocatalytic activity of MoS_2_/Cd*_x_* Zn_1−_ *_x_* S is illustrated in Figure 7F,G. The evolution rate of H_2_ was observed to increase first and subsequently diminish with the increasing Cd content in the photocatalyst. This phenomenon could be elucidated by the reduction of the band gap and photooxidation capability caused by excessive Cd. The stability of Cd*_x_* Zn_1−_ *_x_* S was also assessed, and it showed excellent robustness for four cycles of consecutive 20‐h irradiation. The organic products from the photocatalytic conversion of PET were determined by ^1^H NMR (Figure 7H,I). Besides, the molar ratio of Cd/Zn of the Cd*_x_* Zn_1−_ *_x_* S can greatly affect the product selectivity. These studies revealed the application of photocatalysts with modifiable band gap structures, such as plastic photoreforming over Cd*_x_* Zn_1−_ *_x_* S.

Distinct from nanoparticle and nanosheet photocatalysts, Du and coworkers realized the upgrading of PET, PLA, and PE under visible light irradiation and mild conditions over the MoS_2_‐Tipped CdS nanorods (MoS_2_/CdS) photocatalysts [167]. The MoS_2_ was tipped at one end of CdS nanorods to create a heterojunction, wherein the photogenerated electrons are tended to accumulate on MoS_2_ for favoring the reduction reaction. Meantime, the photoinduced holes left on the CdS were responsible for plastic upgrading. Such a structure with the metal chalcogenides tipped at 1D semiconductors can conduce to ultrafast charge transfer and efficient separation of electron–hole pairs through spatially localized deeper energy states [168, 169, 170, 171]. Upon light illumination, the pretreated PLA and PET with 10 m KOH pretreatment transformed into H_2_ with evolution rates of 6.68 ± 0.01 and 3.90 ± 0.07 mmol g^−1^ h^−1^, respectively. Interestingly, instead of using alkaline pretreatment, PE underwent an acid pretreatment with nitric acid at 180°C for 5 h to convert into short‐chain carboxylic acids (e.g. succinic acid, formic acid, and glutaric acid). The photoreforming of those carboxylic acids could not only generate H_2_, but also produce other gaseous hydrocarbons, including methane, ethane, propane, and n‐pentane. Besides, other aqueous products from PLA, PET, and PE, such as acetate, formate, methanol, and glycolate, were detected using ^1^H NMR spectroscopy. Thereafter, the primary steps for PET, PLA, and PE photoreforming were summarized as follows:

PET

(11) $$C_{2}H_{6}O_{2}+11\;OH^{-}+8\;h^{+}\underset{hv,{\mathrm{MoS}}_{2}/\mathrm{CdS}}{\to }{\mathrm{CO}}_{3}^{2-}+{\mathrm{HCOO}}^{-}+8\;H_{2}O$$

(12) $$C_{2}H_{6}O_{2}+3\;{\mathrm{OH}}^{-}+4\;h^{+}+2\;e^{-}\underset{hv,{\mathrm{MoS}}_{2}/\mathrm{CdS}}{\to }{\mathrm{CH}}_{3}{\mathrm{COO}}^{-}+3\;H_{2}O$$

PLA

(13) $$C_{3}H_{5}O_{3}^{-}+14\;{\mathrm{OH}}^{-}+10\;h^{+}\underset{hv,{\mathrm{MoS}}_{2}/\mathrm{CdS}}{\to }2\;{\mathrm{CO}}_{3}^{2-}+{\mathrm{HCOO}}^{-}+9\;H_{2}O$$

(14) $$C_{3}H_{5}O_{3}^{-}+4\;{\mathrm{OH}}^{-}+H_{2}O\underset{hv,{\mathrm{MoS}}_{2}/\mathrm{CdS}}{\to }2\;{\mathrm{CO}}_{3}^{2-}+{\mathrm{HCOO}}^{-}+5\;H_{2}$$

PE

(15) $$C_{5}H_{8}O_{4}+6\;H_{2}O+17\;h^{+}+e^{-}\underset{hv,{\mathrm{MoS}}_{2}/\mathrm{CdS}}{\to }2\mathrm{HCOOH}+3\;{\mathrm{CO}}_{2}+16\;H^{+}$$

(16) $$C_{6}H_{10}O_{4}+8\;H_{2}O+23\;h^{+}+e^{-}\underset{hv,{\mathrm{MoS}}_{2}/\mathrm{CdS}}{\to }2\mathrm{HCOOH}+4\;{\mathrm{CO}}_{2}+22\;H^{+}$$

**FIGURE 8:**
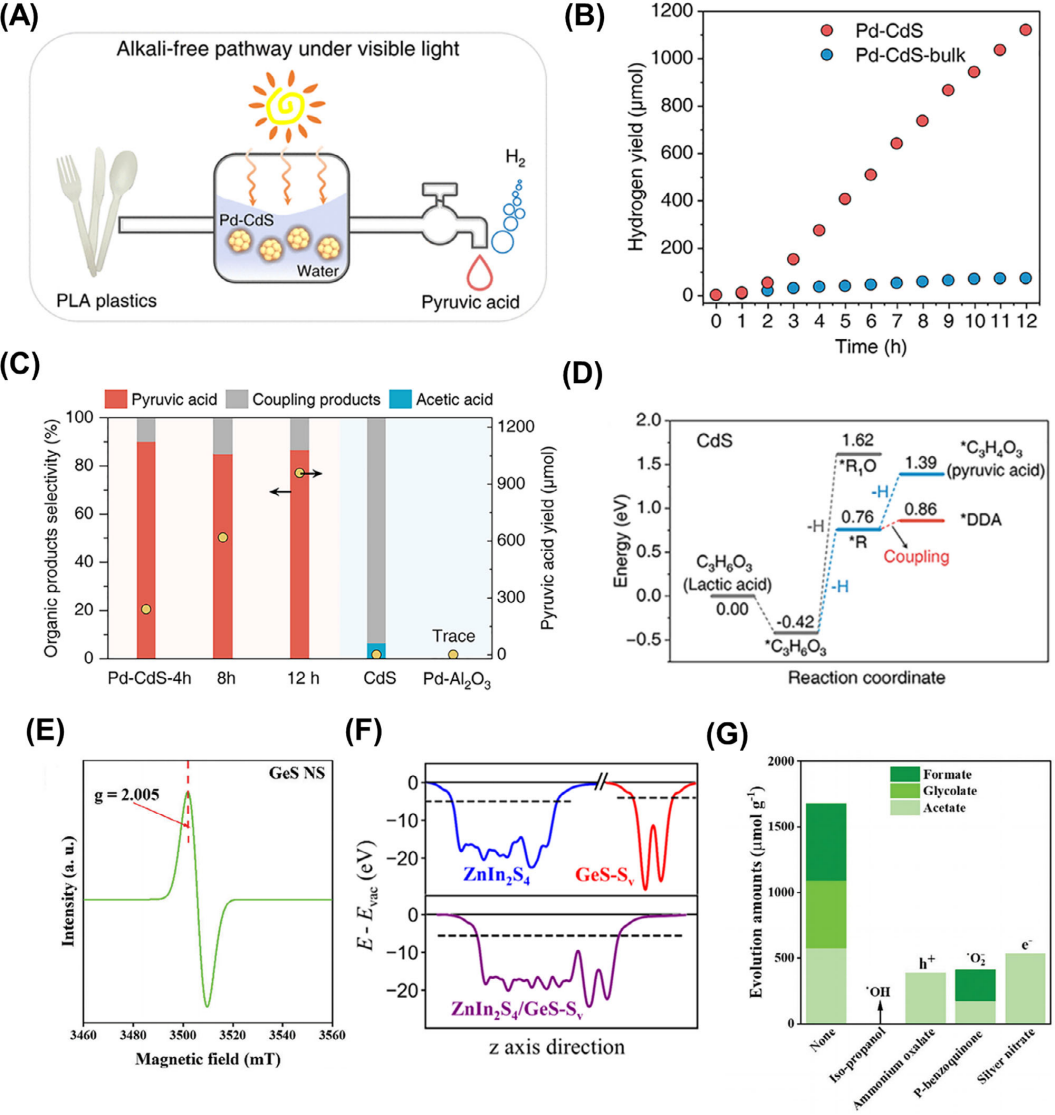
(A) Scheme of alkali‐free photoreforming of PLA using Pd‐CdS. (B) H_2_ evolution rate over Pd‐CdS and Pd‐CdS‐bulk after 12 h visible light exposure. (C) Liquid product selectivity and production over Pd‐CdS. (D) Energy profiles of different reaction routes for lactic acid oxidation through DFT calculations [57]. Reproduced with the permission of Miao et al. [57] Copyright 2024, American Chemical Society. (E) ESR spectrum of 2D S vacancy GeS nanosheet. (F) Electrostatic potential profiles in *z*‐axis direction of 2D/2D S_v_‐GeS/ZnIn_2_S_4_. (G) Products generated after scavenger tests [62]. Reproduced with the permission of Talebian‐Kiakalaieh et al. [62] Copyright 2025, Wiley‐VCH.

Aiming to avoid using the chemical pretreatment, Miao et al. have utilized an alkali‐free pretreatment approach and achieved the selective generation of pyruvic acid from PLA over a Pd‐CdS photocatalyst under visible light illumination (Figure 8A) [57]. By using hydrothermal treatment for PLA granules, Pd‐CdS in pretreated PLA generated a H_2_ and pyruvic acid yield of 1121 and 958.7 µmol after 12 h of irradiation (Figure 8B,C). With the nature of Pd to activate hydroxyl groups and initiate dehydrogenation reaction, a steady selectivity of PLA to pyruvic acid by Pd‐CdS was observed after succeeding photoreforming reaction (i.e. 4 h: 90.2%, 8 h: 85.4%, and 12 h: 86.8%), showcasing the desirable C─H bond cleavage of lactic acid, as evidenced by the density functional theory (DFT) results (Figure 8D). Recently, defect engineering has gained increasing research interest in waste photoreforming due to its intrinsic properties, such as more available active sites, modulated electronic properties, surface reconstruction etc. [52, 62, 172]. For example, Talebian and his group have introduced a 2D/2D S‐vacancy GeS/ZnIn_2_S_4_ hybrid to upcycle raw PET plastic directly into commodity chemicals in seawater by mimicking the natural environment (i.e. sunlight, air, and seawater) [62]. The direct upcycling rate of PET plastic to monomers and organic chemicals (i.e. ethylene glycol, terephthalic acid, formate, acetate, and glycolate) reached up to 13.317 mmol g^−1^ under natural conditions. The sulfur vacancy in GeS was confirmed by electron spin resonance (ESR) spectroscopy with *g* = 2.005, exhibiting a better capturability of electrons (Figure 8E). The role of sulfur vacancy in GeS nanosheet was also verified by the DFT calculation, which showed that the defects can promote the accumulation of electrons on GeS, leading to rapid charge separation and enhanced photocatalytic properties (Figure 8F). Furthermore, hydroxyl radicals and singlet oxygen were the main active species by performing the scavenger tests (Figure 8G).

Unfortunately, the previously reported photocatalysts, such as TiO_2_, faced the issues of morphology alteration in concentrated alkaline solutions at high temperature [173], while CdS photocatalysts contain the highly toxic Cd element [174]. Therefore, developing an economical photocatalyst that is more environmentally benign and efficient than that of the existing options is a necessity to address these issues. In light of this, Nagakawa and Nagata constructed a Cu, In‐doped ZnS (CIZS) photocatalyst from ZIF‐8 to obtain hydrogen from lignocellulosic biomass under simulated solar light [174]. CIZS exhibited a highly ordered crystalline structure compared with that of obtained by solvothermal method, which led to higher catalytic activity. Specifically, the higher crystallinity of CIZS can contribute to fewer recombination centers, thus displaying better photocatalytic activity. Under simulated sunlight with concentrated alkaline solution (10 m NaOH) at 70°C, CIZS successfully photoreformed α‐cellulose to yield H_2_ and organic products such as 5‐hydroxymethylfurfural, monosaccharides, and organic acids. Nevertheless, the apparent quantum yield of CIZS approached to zero near 420 nm, while the quantum yield was 27.94% at 360 nm and 9.58% at 380 nm, which implied that this photocatalyst only responded to UV light, manifesting its low practical potential. All in all, the low redox abilities and stabilities of metal sulfides can be enhanced by different modification strategies, such as element doping and the construction of heterojunction systems, making metal sulfides a fascinating photocatalyst for photocatalytic applications using visible light. The interdependent contributions of various modifications are vital to unveil the research gaps in metal sulfide‐based photocatalysts to offer a thorough understanding for designing a highly efficient semiconductor.

### 3.3 Metal‐Free Photocatalysts

The utilization of metal‐free graphitic carbon nitride (g‐C_3_N_4_) with ample sources for photocatalytic H_2_ production was first reported by Wang et al. in 2009 [175]. Owing to the advantageous properties such as cost‐effectiveness, non‐toxicity, alkaline stability, adequate band positions, and ability to harvest visible light, the metal‐free g‐C_3_N_4_ has attracted considerable interest from research community for various applications (i.e. biomass photoreforming, CO_2_ reduction, water splitting and organic contaminants degradation) [175, 176, 177, 178, 179, 180, 181, 182]. However, g‐C_3_N_4_ possesses some drawbacks such as low photon efficiency and small surface area, which can result in lowering the photocatalytic activity of g‐C_3_N_4_. To this end, exploiting a strategy to improve the photocatalytic activity of g‐C_3_N_4_ is desirable. Several researchers have performed a variety of modification approaches for g‐C_3_N_4_ to overcome the drawbacks. Overall, these approaches can be partitioned into the following types: (a) non‐metal element (e.g. C, N, P, S, and O) doping [183]; (b) copolymerization [184]; (c) surface modification [185]; (d) morphology control [186]; and (e) defect engineering [138].

For instance, Reisner et al. utilized cyanamide‐functionalized C_3_N_4_ (^NCN^CN*_x_*) photocatalysts with the presence of Pt, MoS_2_, and Nibis(diphosphine) (NiP) as cocatalysts to photoreform lignocellulosic biomass and produce H_2_ under mild conditions [47]. The significance of surface functionalization for the photoreforming process is underlined by the diminished performance of bulk ^NCN^CN*_x_* (1.91 ± 0.07 µmol_H__2_) and negligible activity of unfunctionalized ^NH^_2_CN*_x_* (0.13 ± 0.04 µmol_H__2_) after ultraviolet‐visible irradiation for 4 h [187]. This notable difference in the catalytic activity can be attributed to the outstanding oxidation ability of ^NCN^CN*_x_* due to a more deepened valence band position and enhanced hole transfer to the hole scavenger through cyanamide moieties [187, 188, 189]. In addition, under mildly acidic solution, 4‐MBA (cellulose substrate) was successfully photoreformed into H_2_ with an efficient rate of 39,310 ± 1970 µmol g_cat_^−1^ h^−1^. Also, the oxidation aqueous products, such as formate and polysaccharides, were observed utilizing ^13^C NMR spectroscopy. The oxidation reaction was occurred through direct pathway because of strong interaction between ^NCN^CN*_x_* and substrates, where the holes on ^NCN^CN*_x_* possess insufficient oxidation ability to generate ROS [135, 187, 188].

**FIGURE 9:**
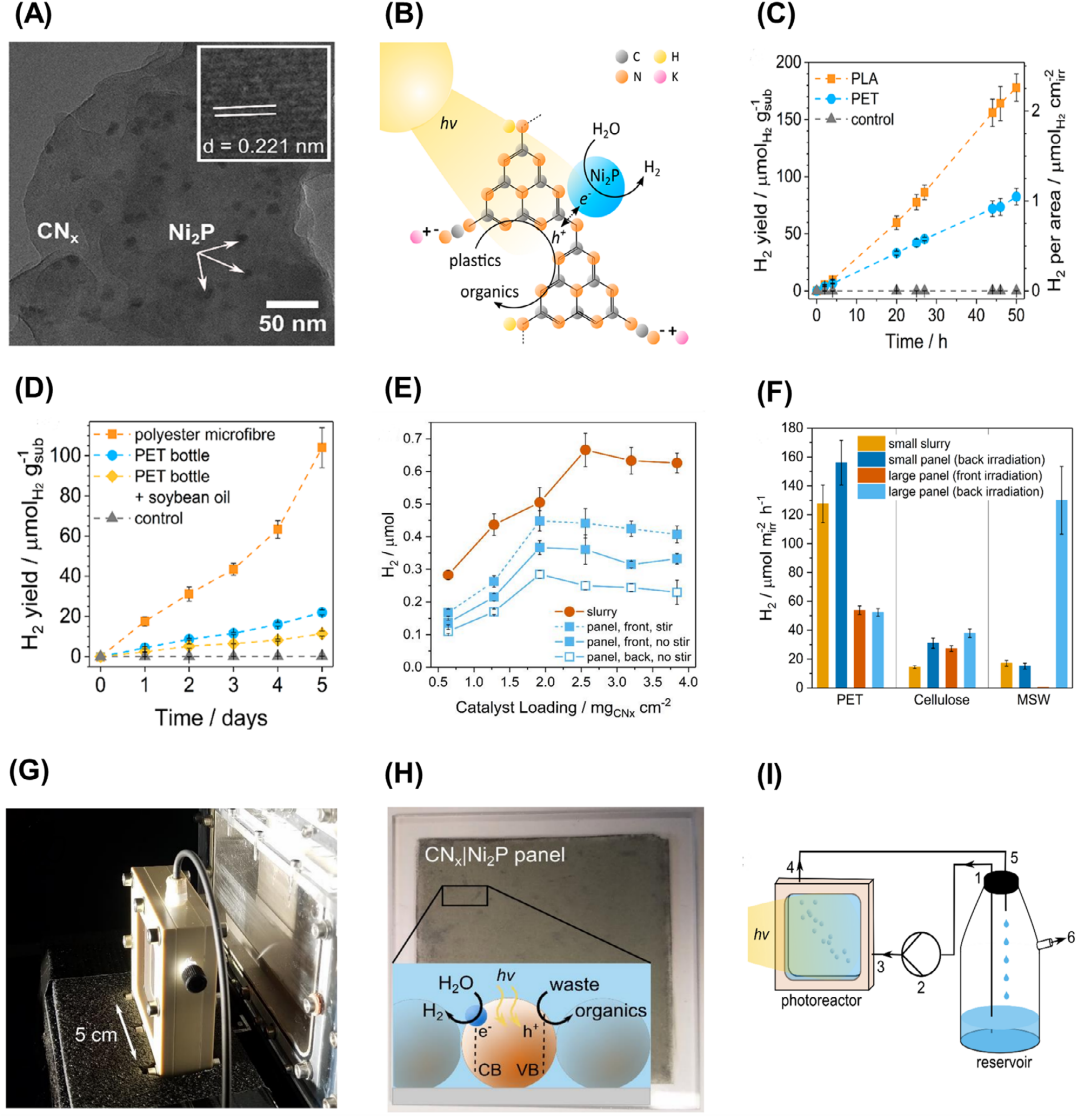
(A) TEM image of CN*_x_* |Ni_2_P with the inset showing the lattice spacing of Ni_2_P. (B) Scheme of plastic photoreforming process using CN*_x_* |Ni_2_P catalysts. (C) H_2_ evolution stability test of CN*_x_* |Ni_2_P using PET and PLA. (D) Long‐term photoreforming of microfibers, PET bottles, and contaminated PET bottles. [48] Reproduced under the terms of the Creative Commons CC BY license [48]. Copyright 2019, American Chemical Society. (E) Optimization of the catalyst loading for H_2_ production. (F) Comparison of small slurry, small‐panel, and different configurations of large‐scale panels for PET photoreforming. (G) Image of large‐scale panels (H) Scheme of photosynthetic process over CN*_x_* |Ni_2_P panel. (I) Image of large‐scale panel experiments in flow [66]. Reproduced with the permission of Uekert et al. [66] Copyright 2020, Wiley‐VCH.

In another work, Reisner and coworkers employed again the ^NCN^CN*_x_* with nickel phosphide (CN*_x_* |Ni_2_P) as cocatalysts for PET and PLA upgrading [48]. Previously, Ni_2_P has been used with ^NH^_2_CN*_x_* and sacrificial agent (triethanolamine) for photocatalytic H_2_ production and possesses high potential for plastic photoreforming due to its chemical stability and good H_2_ evolution activity [190, 191, 192, 193]. The uniform distribution of Ni_2_P nanoparticles on CN*_x_* were observed using TEM (Figure 9A). During the photocatalysis reaction, the photoexcited electrons transferred to Ni_2_P for H_2_ production, while the plastics served as sacrificial agents and were photooxidized to organic compounds (Figure 9B). The stability of CN*_x_* |Ni_2_P was evaluated by the long‐term photoreforming of PLA and PET, generating a constantly increasing evolution rate of H_2_ for 50 h of irradiation (Figure 9C). To access the practical potential of CN*_x_* |Ni_2_P photocatalysts in reality, another long‐term study of PET bottles, food‐contaminated PET, and polyester microfibers was carried out, generating H_2_ with evolution rates of 22, 11.4, and 104 µmol g^−1^ after irradiation for 5 days, respectively (Figure 9D). In fact, the mechanism of solar‐driven upcycling of waste polymer began with the depolymerization step via hydrolysis, followed by the redox reactions of producing H_2_ and CO_2_ gases. The main steps of PET and PLA photoreforming over CN*_x_* |Ni_2_P catalyst are summarized by the Reisner group, which is shown below:

**Table:** 

(1) PET depolymerization	C_10_H_8_O_4_ + 2 H_2_O $\underset{\mathrm{Alkali}}{\to }$ C_8_H_6_O_4_ + C_2_H_6_O_2_
(2) Photoreduction of ethylene glycol	C_2_H_6_O_2_ + 2 H_2_O $\underset{hv,CN_{x}}{\to }$ 5 H_2_ + 2 CO_2_
(3) PLA depolymerization	C_3_H_4_O_2_ + H_2_O $\underset{\mathrm{Alkali}}{\to }$ C_3_H_6_O_3_
(4) Photoreduction of lactic acid	C_3_H_6_O_3_ + 3 H_2_O $\underset{hv,{\mathrm{CN}}_{x}}{\to }$ 6 H_2_ + 3 CO_2_

On the other hand, Reisner et al. proposed a photocatalyst panel by immobilizing CN*_x_* |Ni_2_P particles on a frosted glass to conquer the challenges in upscaling the nanoparticle photocatalyst systems (e.g. catalyst sedimentation, obstruction of light by the mixture, and troubles in recycling) [66]. The hydrogen evolution rate continued to increase with low catalyst loadings and plateaued with higher loadings (Figure 9E). It was noted that the small panels with 1 cm^2^ yielded a relatively high H_2_ evolution rate to the slurry containing solid wastes (Figure 9F). Also, the panel size was enlarged to 25 cm^2^ to compare the configuration of front and back irradiation in large panels (Figure 9G, H). The back irradiation was observed to have better H_2_ yield and could be applied in contaminated colored solutions. Consequently, photoreforming over the catalyst panels in flow could provide several benefits such as easy preparation, cost‐effectiveness, and continuous production of H_2_ and high‐value organic products (Figure 9I) [66].

Additionally, Reisner et al. reported a tandem process that combined plastic oxidation with photocatalytic routes to transform PE into fine chemicals [90]. In the polymer oxidative process, PE was first degraded to dicarboxylic acids (i.e. succinic acid and glutaric acid) by dilute HNO_3_ as oxidants. The dicarboxylic acids were then transformed into gaseous hydrocarbons by photocatalysis over ^NCN^CN*_x_* or TiO_2_. Distinct from the batch system, a photocatalytic flow setup that consisted of a pump, reservoir, photocatalyst panel, and photoreactor was prepared to study its practical application. With this flow system, ethane, propane, ethylene, and propylene could constantly produce over P25|Pt and ^NCN^CN*_x_* |Pt photocatalysts during 3 days of reaction time, wherein the product selectivity was highly dependent on the type of photocatalyst. For TiO_2_|Pt, ethane and propane were observed as the primary products, while ethene and propene were detected as the primary oxidation products on ^NCN^CN*_x_* |Pt. Consequently, this two‐step chemical‐photocatalytic method can utilize abundant sunlight to efficiently convert PE into high‐value and easily separable gaseous products.

Different from binary photocatalysts, Huang et al. recently reported a ternary carbon nitride‐carbon nanotubes‐NiMo (CN‐CNTs‐NiMo) photocatalyst for converting PET into H_2_ under benign conditions [194]. Although NiMo could act as cocatalyst to overcome the limited photocatalytic activity of CN aroused by the rapid recombination of charge carriers, the sluggish transfer of photogenerated electrons from CN to NiMo was another problem required to be resolved for photoreforming process of PET. In fact, the interfacial engineering of semiconductors by introducing the electron transfer layer could enhance the interfacial electron transfer, leading to improve photocatalytic activity [188]. Therefore, CNTs with beneficial properties (e.g. large surface areas, excellent electron mobility [195], and outstanding alkaline stability [196, 197]) could couple with CN to offer an ideal case for enhancing the activity of PET photoreforming as CNTs could use as a superior charge transfer layer to offer an efficient electron transport network via π─π^*^ conjugation [198]. The π─π^*^ conjugation between CN and CNTs ameliorated the behavior of carrier and alleviated the transfer barrier of electrons, thus improving the efficiency of photoreforming reaction. As a result, CN‐CNTs‐NiMo manifested a remarkable H_2_ yield in PET photoreforming, which was 14 folds greater than that of CN.

Another notable example was carried out by Yan et al. who prepared a ternary Ni*_x_* Co_1−_ *_x_* P/rGO/g‐C_3_N_4_ hybrids as photocatalysts to photoreform PLA in alkaline aqueous medium [199]. Metal phosphides are often applied as cocatalyst to improve the photocatalytic activity of g‐C_3_N_4_ due to their good affordability. Compared with single‐metal phosphides, bimetallic phosphides were more advantageous in improving the charge transfer owing to the lower overpotential for H_2_ production [193, 200, 201]. Whereas rGO acted as electron transport medium in this system as it could store and transfer electrons to the catalytic sites [202, 203]. Upon visible light illumination, the photoexcited electrons migrated to Ni*_x_* Co_1−_ *_x_* P via the following two routes: 1) Schottky junction between Ni*_x_* Co_1−_ *_x_* P and CN; and 2) through rGO as an electron transport layer. Then, the photogenerated electrons in Ni*_x_* Co_1−_ *_x_* P would reduce the proton to H_2_ [204, 205]. Meanwhile, the photoinduced holes left on Ni*_x_* Co_1−_ *_x_* P/rGO/g‐C_3_N_4_ and hydroxyl radicals would oxidize the pretreated PLA into value‐added products such as formate and acetate. Other than the Schottky junction, Li and his team have successfully prepared an H_5_PMo_10_V_2_O_40_/g‐C_3_N_4_ (VPOM/CN) photocatalyst to realize the selective generation of formic acid under an oxygen‐rich environment by transforming PE, PP, and PVC [206]. The yield rate of formic acid from PE could reach up to 24.66 µmol g^−1^ h^−1^ by using VPOM/CN, which is 262‐fold higher than the pristine CN. The outstanding improvement in the photoreforming performance was attributed to the successful construction of the Z‐scheme heterostructure. Such a heterostructure allowed a better charge transfer ability by shifting the electrons from the protonated CN to the negatively charged VPOM. With the presence of an internal electric field, the lifetime of the charge carriers was prolonged from 220.12 to 389.74 ps.

**FIGURE 10:**
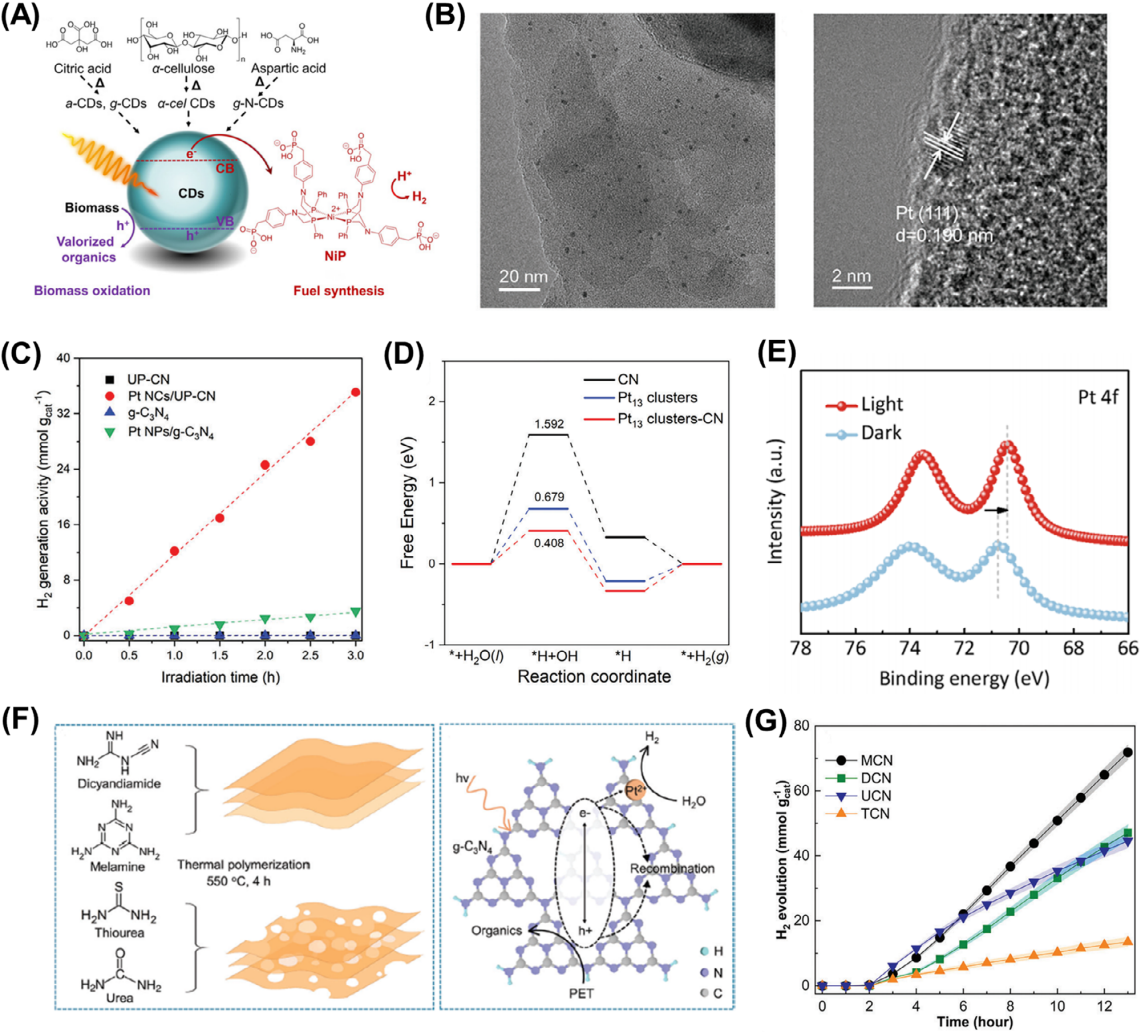
(A) Scheme of utilization of NiP/CDs in xylan and α‐cellulose photoreforming [207]. Reproduced under the terms of the Creative Commons CC BY license [207]. Copyright 2020, Wiley‐VCH. (B) HRTEM images of UP‐CN anchored with Pt nanoclusters. (C) H_2_ yield of photoreforming for PET over UP‐CN/Pt. (D) Free energy profiles for solar‐driven H_2_ production process using CN/Pt [208]. Reproduced with the permission of Hu et al. [208] Copyright 2024, Wiley‐VCH. (E) XPS spectra of Pt 4f under dark and light [56]. Reproduced with the permission of Li et al. [56] Copyright 2024, American Chemical Society. (F) Schematic diagram of g‐C_3_N_4_ synthesis from different carbon‐nitrogen‐rich precursors. (G) H_2_ production rate of g‐C_3_N_4_ derived from different precursors [209]. Reproduced with the permission of Nguyen et al. [209] Copyright 2023, Wiley‐VCH.

Aiming to achieve biomass valorization, Achilleos et al. prepared homogeneous carbon dots (CDs) coupled with NiP for the photoreforming of xylan and α‐cellulose into carbon‐rich organics (e.g. C_6_H_12_O_6_, C_6_H_10_O_5_, and hydroferulic acid derivatives) while evolving H_2_ fuels [207]. Carbon dots and NiP were employed as a light absorber and H_2_ evolution cocatalyst, respectively (Figure 10A). With the aid of NiP, the H_2_ yield of xylan and α‐cellulose reached up to 5.0 ± 0.2 and 3.6 ± 0.3 µmol, respectively, which is similar to the heterogeneous CDs/NiP in previous reported work. Recently, the ultrathin porous g‐C_3_N_4_ (UP‐CN) has shown great interest from researchers in upcycling PET plastic with its exceptional catalytic activity in an alkaline environment. Herein, Hu and his team performed the photoconversion of polyester using UP‐CN integrated with Pt nanoclusters [208]. Due to the presence of Pt and porous structure, the photocatalytic performance of Pt/UP‐CN could generate a H_2_ yield of 11.69 mmol^−1^ g^−1^ h^−1^ for PET photoreforming under an alkaline environment (Figure 10B,C). Such modifications on CN improved the interaction between the substrate and catalyst and offered more accessible active sites for redox reactions. As evidenced by the DFT (density functional theory) calculation, anchoring the Pt nanoclusters on UP‐CN significantly alleviated the thermodynamic barriers of the water splitting reaction to 0.408 eV compared to pristine UP‐CN (1.59 eV) and Pt (0.68 eV), as shown in Figure 10D. From the work of Zhang and his team, the characteristic peaks of Pt 4f in XPS spectra shifted negatively under light irradiation compared to in the dark, implying the migration of electrons from CN to Pt for realizing a low charge recombination rate (Figure 10E) [56]. On the other hand, Nguyen et al. successfully utilized different precursors, including melamine, dicyanamide, thiourea, and urea, to synthesize CN in order to investigate the structure‐activity relationship for PET photoreforming (Figure 10F) [209]. The melamine‐derived CN could exhibit a remarkable H_2_ generation rate of 7.33 mmol^−1^ g^−1^ h^−1^ with the aid of Pt as a reductive cocatalyst (Figure 10G). With these contexts, the synergy of Pt and CN could lower the energy barrier for H_2_ evolution and promote rapid charge separation.

Overall, carbon‐based photocatalysts for solid waste photoreforming are an ideal alternative to metal‐based photocatalysts with their great potential of a remarkable surface‐to‐volume ratio and good light responsivity, resulting in favorable reactivity. However, these metal‐free photocatalysts still face the obstacle of achieving the reduction potential for high‐efficiency solar fuel generation in their pristine form. Thus, the development of modification strategies should always be in line with the fundamental study of carbon‐based photocatalysts due to their low quantum efficiency for photocatalysis.

### 3.4 Other Catalysts

**FIGURE 11:**
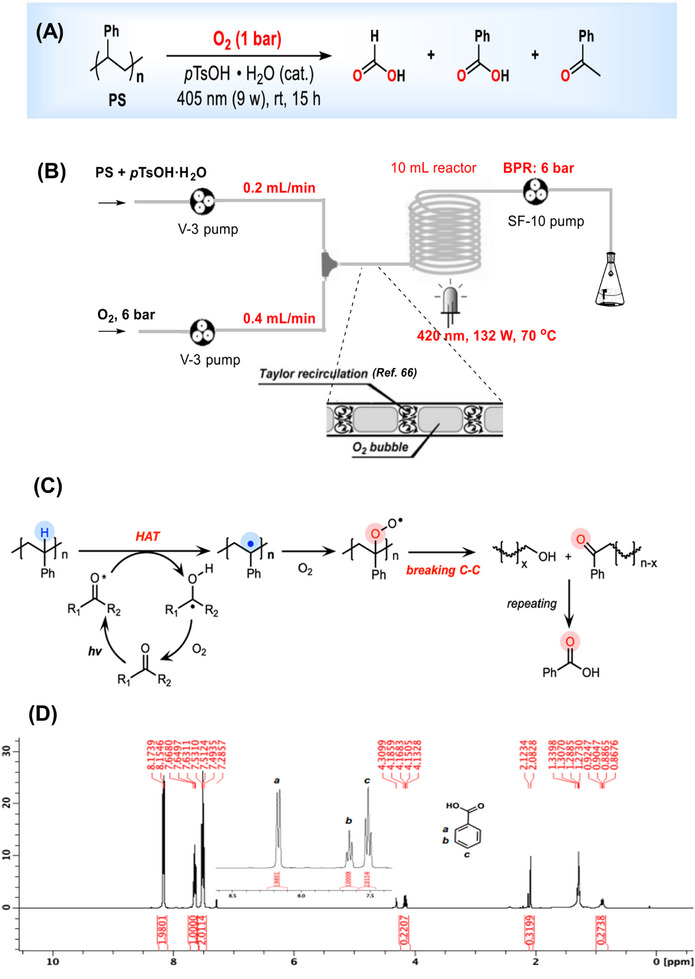
(A) Scheme of acid‐catalyzed reaction to degrade PS, (B) Optimized set and conditions of flow oxidation of PS [214]. Reproduced under the terms of the Creative Commons CC BY license [214]. Copyright 2022, American Chemical Society. (C) Scheme of photocatalytic deconstruction of PS by C─H bond oxidation on a tertiary sp^3^ carbon, (D) ^1^H NMR spectra for benzoic acid from real‐life PS deconstruction [216]. Reproduced under the terms of the Creative Commons CC BY license [216]. Copyright 2022, American Chemical Society.

Besides heterogeneous photocatalysts, photosynthesis from waste into chemicals can also be driven by using homogeneous photocatalysts. Despite its advantages of endowing the processes to take place under mild conditions and more accessible catalytic sites, the homogeneous catalysis also concurrently provides deeper insight into the mechanistic study of the photoreforming process at the microchemistry level to enhance the catalytic reaction [210, 211]. As such, many industrial applications, including the pharmaceutical and fine chemicals industries, employ homogeneous catalysts in the complex synthesis process. [212, 213] Upon absorption of light, the catalysts undergo excitation, initiating the HAT process or generating reactive intermediates such as singlet oxygen, hydroxyl radicals (•OH), or superoxide anions (O_2_^•−^) for catalytic oxidative reactions. Very recently, Xiao et al. reported a solar‐driven selective acid‐catalyzed oxidation reaction to upgrade PS into valuable products under visible light irradiation (Figure 11A) [214]. The catalytic oxidation of PS often faces the issue of long degradation duration, and upgraded products cannot be recycled after use. This technique utilizes the inexpensive and abundant organic or inorganic acids as facile catalysts to oxidatively cleave PS into benzoic acid, acetophenone, and formic acid by singlet oxygen (^1^O_2_) under ambient conditions. Generally, ^1^O_2_ has comparably higher energy (94 kJ/mol) compared with steady‐state O_2_ [215]. Thus, it has the ability to initiate the oxidation of multifarious organic compounds at low temperatures, motivating a wide range of applications such as chemical and biochemical reactions. The system employed 5 mol% p‐toluenesulfonic acid monohydrate (pTsOH·H_2_O) as a homogeneous catalyst and benzene/acetonitrile (C_6_H_6_/CH_3_CN, 1:1) as a solvent to give optimum yields. Under visible light irradiation for 15 h, the yield of acetophenone, benzoic acid, and formic acid could achieve up to 2, 50, and 67%. Moreover, the upscaling of this protocol was successfully realized by the degradation of PS in flow utilizing the continuous flow microreactor (Figure 11B).

In another work, Reisner and his group prepared a hydrogen atom transfer (HAT) photocatalyst for photocatalytic degradation of PS via C─H bond oxidation [216], which was enlightened by the selective bond scission in short‐chain hydrocarbons. This strategy used nontoxic, inexpensive fluorenone (aromatic ketone) as a photocatalyst and ethyl acetate as a solvent to deconstruct PS into benzoic acid, benzaldehyde, ethyl benzoate, phenylglyoxylic acid, and acetophenone under ambient pressure and 50°C. The reaction proceeded as follow (Figure 11C) [217]: (1) the generation of PS and ketyl radicals during the HAT process between the substrate and excited photocatalyst; (2) the regeneration of photocatalyst by the reduction of oxygen to hydrogen peroxide or/and water; (3) the inducement of C─C bond scission at the PS backbone by the absence of second H_2_ to yield the products from degradation. Different from the acid‐catalyzed reaction, 95% of the photocatalyst in this system can be recycled from 1 day mixture. Then, the recycled fluorenone still can produce benzoic acid with 34% yield in the second reaction. Also, to examine the practical potential of this system, a feasibility study was performed through a gram‐scale degradation of pure PS and actual PS foam. The yield of benzoic acid from pure PS and PS foam was 40% and 38%, which detected using ^1^H NMR (Figure 11D). This result can show the potential and practicality of this photo‐driven C─H bond oxidation system for a broad range of solid wastes.

On the other hand, the metal catalysts, peroxides, quinones, and benzophenones have been widely used as photoinitiators to facilitate the polymer backbone oxidation through HAT process under UV irradiation [218, 219, 220, 221]. Herein, Stache and Oh reported the use of chlorine radicals to extract electron‐rich hydrogen from PS and produce ROO**·** for C─C bond cleavage under visible light irradiation [222]. As a low‐cost and scalable alternative, FeCl_3_ and other similar compounds could act as catalysts for the generation of chlorine radicals using visible light [223, 224]. The photogenerated chlorine radicals facilitated the abstraction of C─H bond from the backbone of PS, generating a benzylic radical, in which this radical would react with O_2_ and further produce ROO**·**. HAT process provided a peroxide (ROOR), and the reduction of ferrous ion gave the ROO**·** and regenerated the ferric ion. After a series of β‐scission and oxidation, benzaldehyde was generated from an oxygen‐centered radical, whereas a primary radical was also regenerated. Eventually, the final product of the photooxidation process was benzoic acid from the oxidation of benzaldehyde. To study the feasibility and scalability of this method, a dual phase flow system was employed to degrade real world PS (Styrofoam) in gram scale. The benzoic acid and benzoyl products were detected using ^1^H NMR with yield of 10.8 and 20.4 mol %, respectively.

However, the oxidative cleavage of PS to benzoic acid employing a FeCl_3_ catalyst had low product selectivity and long reaction time, which could restrain its practical applications [222, 225]. Also, there is a big challenge for the photocatalytic conversion of plastic wastes into low molecular weight aromatics such as benzene and toluene. To this end, designing an efficient method for the transformation of plastic waste is highly desirable. For this purpose, Das et al. proposed the generation of selective carbon‐centered radicals on the phenyl group bearing carbon in PS via HAT process to yield oxidized products, such as benzoic acid, with the presence of a metal‐free photocatalyst [226]. By means of tandem fashion, the benzoic acid could be further converted into low molecular weight liquid bromosuccinimide, which was used as the photocatalyst to produce bromine radicals for the abstraction of a hydrogen atom from the C─H bond in PS. Meanwhile, ethyl acetate and CF_3_SO_2_Na were employed as solvents and pentacoordinate sulfide‐producing reagents. In contrast with the previous report, with optimal conditions, the yield of benzoic acid in this system could reach to 73 % upon light illumination (390 nm) for 16 h. A gram‐scale reaction was performed to convert the real‐life PS (e.g. plastic cups and foam) into benzoic acid, where eventually large quantities (>60 %) of benzoic acid were produced, showcasing its feasibility and scalability. Furthermore, the benzoic acid was successfully converted into various value‐added aromatic building blocks and bioactive molecules through a tandem fashion, which further exhibited the practical utility of this system. In short, the scalability of homogeneous photocatalysis seems to be more facile than that of heterogeneous photocatalysis, yet the recycling issue of homogeneous photocatalysts can be a big challenge when upscaling the flow system.

## 4 Application of External‐Field‐Assisted Process in Photoreforming

Since the pioneer study of Honda and Fujishima using photoelectrochemical (PEC) system to generate hydrogen over a TiO_2_ electrode under light irradiation [266], external‐field‐assisted processes have been extensively exploited in catalysis field, especially photocatalysis, toward the production of fuels or high‐valued commodity chemicals. In the past decades, photocatalysis has advanced into a relatively mature and complete system which involves multidisciplinary studies, including photo/electrochemistry, material design, catalytic chemistry, and semiconductor physics [267]. Nonetheless, there are intractable bottlenecks in the state‐of‐the‐art photocatalysts, including deficient light harvesting ability, poor stability, and low selectivity, that makes a challenge to enhance photocatalytic efficiency in this field of research. For example, the rapid recombination of charge carriers that always occurred in common photocatalysts was attributed by their intrinsic properties [268]. Moreover, the solid waste degradation process is unfavorable as it is nonselective and often co‐exists with several side reactions, resulting in uncontrollable product generation [44]. To this end, introducing the external fields into the photocatalytic system can be an effective and controllable strategy to ameliorate the photocatalytic activities and impact the performance of photocatalyst. In fact, there are several external fields that can be applied into the field of photocatalysis including thermal, electrochemical, piezoelectric, pyroelectric, and biocatalysis [269, 270, 271, 272, 273, 274, 275, 276]. Such external driving sources can bring additional energy to photocatalytic systems to facilitate the electron–hole pairs separation and enhance the charge mitigation, hence boosting the current photocatalytic system. A summary of recent study on external‐field‐assisted process in solid waste photoreforming is presented in Table 3.

**TABLE 3:** Summary of external‐field‐assisted photocatalysis on solid waste valorization.

Catalyst	Field	Solid waste	Reaction conditions	Products	Performance	Ref
Cu_30_Pd_70_|Pervoskite|Pt	Photoelectro	PET powder PET bottle Cellulose	Solar light irradiation (AM 1.5G, 100 mW cm^−2^), 10 h	H_2_ Gluconic Acid Glycolic acid	H_2_ yield from PET powder: 737 µmol cm^−2^ H_2_ yield from PET bottle: 705 µmol cm^−2^ H_2_ yield from cellulose: 438 µmol cm^−2^ Product selectivity: 60–90%	[283]
Fe_2_O_3_/Ni(OH)*_x_*	Photoelectro	PET	Visible light irradiation (10‐100 mW cm^−2^)	H_2_ Formic acid	Faradaic efficiency of formic acid: ∼95%	[310]
CNT/NiP	Photoelectro	PET	Solar light irradiation (AM 1.5G, 100 mW cm^−2^)	H_2_ Formic acid	Faradaic efficiency of formic acid: ∼95%	[311]
Ti‐doped α‐Fe_2_O_3_	Photoelectro	Polyimide	Solar simulator (AM 1.5G, 100 mW cm^−2^)	H_2_ Formic acid Acetic acid Succinic acid	Selectivity toward formic acid: ∼28% Selectivity toward acetic acid: ∼41% Selectivity toward succinic acid: ∼22% Overall faradaic efficiency: 91.6%	[284]
Ni‐Pi/ α‐Fe_2_O_3_	Photoelectro	PET	300 W Xe lamp (AM 1.5G, 100 mW cm^−2^), 20 h	H_2_ Formic acid Glycolic acid	H_2_ yield from PET: ∼410 µmol cm^−2^ Formic acid yield: ∼230 µmol cm^−2^ Glycolic acid yield: ∼40 µmol cm^−2^ Faradaic efficiency: ≥95%	[312]
BiVO_4_	Photoelectro	Tartaric acid	300 W Xe arc lamp (AM 1.5G, 100 mW cm^−2^), 100 min	H_2_	H_2_ yield: ∼37 µmol Faradaic efficiency: ∼94%	[287]
Zr‐doped α‐Fe_2_O_3_ with redox enzymes (r*Aae*UPO, GDH, *Ts*OYE)	Photoelectro‐bio	PET	Xe lamp (AM 1.5G, 100 mW cm^−2^)	Formic acid Acetic acid Enantio (R)‐1‐phenylethanol l‐glutamate Enantiopure (R)‐2‐methylcyclohexanone	Formic acid yield: 1600 nmol h^−1^ Acetic acid yield: 55 nmol h^−1^ TTN_r_ *_Aae_*_UPO_: 362,000 TTN_GDH_: 144,000 TTN*_Ts_*_OYE_: 1,300	[288]
CNT modified by polydopamine	Photothermal	PET	Simulated sunlight, ambient pressure, 2.5 h	Bis‐2‐hydroxyethyl terephthalate (BHET)	PET degradation: 100% BHET yield: ∼52%	[313]
g‐C_3_N_4_/WP/W SAs	Photothermal	PET	300 W Xe lamp (AM 1.5G)	H_2_	H_2_ yield from PET: 562.5 µmol g_cat_^−1^ h^−1^	[311]
Pt/TiO_2_‐O_v_	Photothermal	Lignin	Solar simulator (AM 1.5G, 100 mW cm^−2^, 473 K)	H_2_	H_2_ yield from lignin: 296.9 µmol	[314]
Ru/TiO_2_	Photothermal	LDPE	Xe lamp (3 W cm^−2^), 30 bar, 220°C, 3 h	C_1_–C_4_ gases C_5_–C_12_ liquid fuels	Selectivity toward C_1_–C_4_ products: ∼15% Selectivity toward C_5_–C_12_ products: ∼82%	[295]
CDPCN with *M.b*	Photobio	PLA rice straw PET glove PUR plastic film PS plastic bag	24 days of illumination	CH_4_	CH_4_ yield from PLA: 1.78 mmol g^−1^ CH_4_ yield from PET: 0.16 mmol g^−1^ CH_4_ yield from PUR: 0.88 mmol g^−1^ CH_4_ yield from PS: 0.17 mmol g^−1^	[306]
Fluorescein‐cage‐NADH clathrate	Photobio	Glucose	300 W Xe lamp	H_2_	H_2_ yield: 226 µL TTN: 15,000	[307]
TiO_2_/Pt with LCC enzyme	Photobio	PET	Solar simulator (AM 1.5G, 100 mW cm^−2^)	H_2_ Formic acid	H_2_ yield from PET: 9.784 mmol m_irr_^−2^ Formic acid yield: 5 µmol	[308]
PVDF/Fe_3_O_4_@g‐C_3_N_4_	Piezoelectric and pyroelectric‐photo	Glucose	Xe lamp (AM 1.5G), 3h	H_2_ Formic acid	H_2_ yield from glucose: 42.3 µmol h^−1^ AQY (full solar spectrum): 12.6%	[309]

The utilization of powder suspension in solid waste photoreforming or common photocatalytic reaction could arouse the difficulties of optimizing the catalyst design, and show poor efficiency and recyclability from remnant solid wastes [277] and PEC system could be an alternative to the solid catalyst powder [278]. Owing to the externally applied potential, the PEC approach in solid waste transformation has mighty potential by minimizing the recombination rate of charge carriers and fully utilizing generated electrons in clean fuel generation. In a PEC system, the basic setup includes the electrolyte solution, a counter electrode, and a working electrode. Different from electrocatalysis, one or more electrodes in PEC system belong to materials that respond to light irradiation, allowing the absorption of photon energy to drive redox reactions. Taking photoanode as an example, the electron–hole pairs will be generated under light irradiation, in which the electron will transfer to the cathode for reduction reaction, and the inverse reaction takes place on the surface of the photoelectrode [279]. Similar processes will occur at the photocathode, while holes are the carriers traveling to the anode for the oxidation reaction. Additionally, it is worth noting that the efficiency of PEC approach is mainly dependent on various factors including the electrode bias, type of electrolyte, and operating current density as well as the light harnessing ability and catalytic activity of semiconductors [280, 281].

**FIGURE 12:**
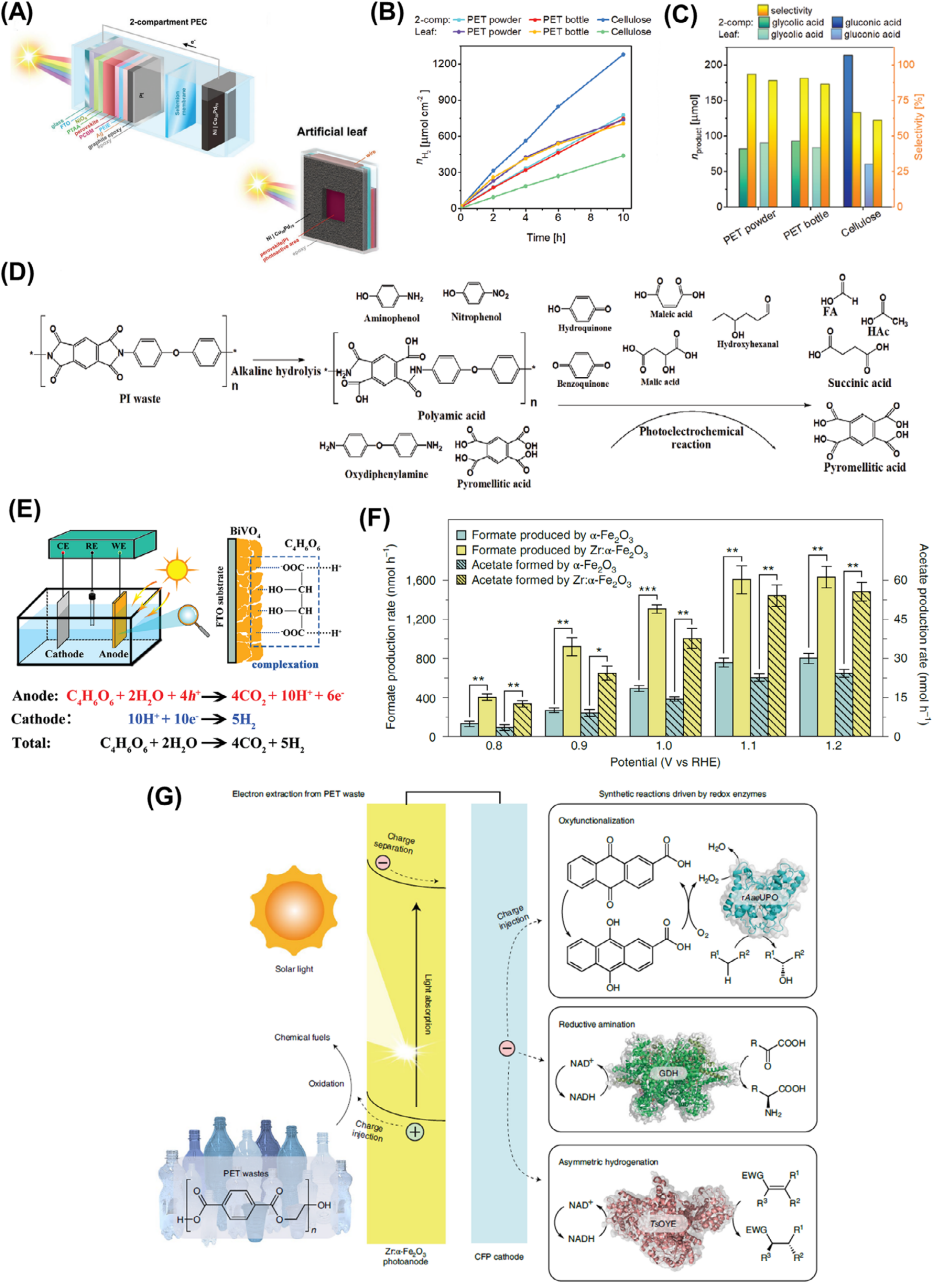
(A) Scheme of Cu_30_Pd_70_|Perovskite|Pt system in two‐compartment and “artificial leaf” configurations, the amount of (B) H_2_ and (C) oxidation products from solid waste and the corresponding selectivity after 10 h of bias‐free measurement [283]. Reproduced under the terms of the Creative Commons CC BY license [283]. Copyright 2021, Wiley‐VCH. (D) Proposed reaction pathway of polyimide upgrading [284]. Reproduced with the permission of Zhao et al. [284] Copyright 2024, Wiley‐VCH. (E) Scheme of BiVO_4_ fuel cell system [287]. Reproduced with the permission of Wang et al. [287] Copyright 2022, Wiley‐VCH. (F) Production rates of formate and acetate driven by α‐Fe_2_O_3_ and Zr:α‐Fe_2_O_3_ photoanodes, and (G) solar‐driven PEC biosynthetic system using PET [288]. Reproduced with the permission of Kim et al. [288] Copyright 2022, Springer Nature.

A tandem fashion with photoanode and photocathode was always found out on PEC water splitting and CO_2_RR to offer adequate driving force for bias‐free system. For example, Jiang et al. paired an n‐type g‐C_3_N_4_ semiconductor with p‐type CuO in a PEC system to enhance the CO_2_‐to‐methanol yield by 4.1 and 2.6 times in comparison to g‐C_3_N_4_/carbon paper and CuO/carbon paper [282]. In spite of that, solid waste oxidation with less energy requirement could avoid the use of tandem configuration with stern requirement and utilize lead halide perovskite as a single light absorber in solar energy conversion. For this purpose, Reisner and his group integrated Cu_30_Pd_70_ alloys (oxidative catalyst) with perovskite|Pt (photoactive material + reductive catalyst) to prepare a bias‐free PEC device to perform photoreforming of soluble biomass and plastic wastes under simulated solar light irradiation [283]. The PEC device was designed to be capable of operating in both two‐compartment and integrated “artificial leaf” configurations (Figure 12A) after combination of perovskite|Pt and Ni foam|Cu_30_Pd_70_. In comparison to both configurations, the photocurrent density of stand‐alone artificial leaf devices in cellulose and pretreated PET solution was lower compared to that in the two‐compartment configuration due to the light absorption in colored solution, light scattering effect, and poisoning of the catalyst. From Figure 12B, both models could give outstanding and promising performance on H_2_ evolution: (a) 2‐compartment: PET powder (776.2 µmol_H__2_ cm^−2^), PET bottle (747.8 µmol_H__2_ cm^−2^), and cellulose (1279.8 µmol_H__2_ cm^−2^), and (b) leaf: PET powder (737 µmol_H__2_ cm^−2^), PET bottle (703 µmol_H__2_ cm^−2^), and cellulose (438 µmol_H__2_ cm^−2^). Meanwhile, the aqueous oxidation products such as gluconic acid and glycolic acid were achieved with approximately 60–215 µmol of yield and 60–90% of product selectivity (Figure 12C).

Recently, Zhao and his team fabricated a hematite α‐Fe_2_O_3_ decorated with Ti as a dopant for H_2_ generation and selective cleavage of benzene rings in polyimide [284]. From Figure 12D, those hydrolyzed intermediates that contained nucleophile groups (e.g. ─NO_2_, ═O, or ─OH) would readily donate electrons to the surface of electron‐deficient Ti‐α‐Fe_2_O_3_, leading to weakening the benzene rings and selectively producing formic acid, acetic acid, and succinic acid [285, 286]. In addition to a new view of generating H_2_ from biomass organic molecules, Huang et al. proposed a PEC fuel cell using BiVO_4_ photoanodes to convert the tartaric acid (C_4_H_6_O_6_) with rich carboxyl and hydroxyl groups into fuels [287]. The strong complexation between the photoanode and C_4_H_6_O_6_ helps construct a strong interaction to form a bridge for charge and energy transfer (Figure 12E). Under simulated solar light irradiation, the photoinduced charge carriers (4e^−^ and 4h^+^) are produced at the BiVO_4_, where the electrons are transferred to the cathode with the aid of applied bias potential from the potentiostat. Meanwhile, the holes left on photoanode were consumed to activate the oxidation of tartaric acid into 6 electrons, 10 H^+^, and 4 CO_2_. Afterward, the electrons from organic oxidation were moved to the cathode and joined the four photogenerated electrons with a total of 10e^−^ to take part in the proton reduction for H_2_ generation, thus providing a paradigm to improve conventional fuel cells.

In another interesting work, Park and coworkers succeeded in making use of two external‐field‐assisted processes in photocatalysis, in which they realized a biocatalytic PEC (BPEC) platform by integrating photoelectrocatalysis and bioredox reaction for real‐world plastic waste conversion under an alkaline environment [288]. In this system, zirconium‐doped α‐Fe_2_O_3_ was applied as a photoanode to transfer electrons from PET hydrolysate, whereas anthraquinone‐2‐carboxylic acid‐bearing carbon fiber paper (AQC/CFP) was employed as a cathode to generate NADH or H_2_O_2_, respectively. Then, several redox enzymes (i.e. NADH‐dependent ene‐reductase: *Ts*OYE, NADH‐dependent L‐glutamate dehydrogenase: GDH, and H_2_O_2_‐dependent unspecific peroxygenase: r*Aae*UPO) were used to catalyze the enzymatic synthetic reaction. In a two‐compartment configuration, the hydrolyzed PET solution was converted into acetate and formate by the photoinduced holes from a single photoactive material, as evidenced by ^1^H and ^13^C NMR analysis. The introduction of Zr dopants into α‐Fe_2_O_3_ semiconductor resulted in an increase in the electron concentration and improved charge separation dynamics, leading to a faster PET reformation than the pristine α‐Fe_2_O_3_ from 0.8 to 1.2 V_RHE_ (Figure 12F). By contrast, the photobiocatalytic site produced organics, including enantiopure (*R*)‐1‐phenylethanol, l‐glutamate, and enantiopure (*R*)‐2‐methylcyclohexanone, and showed corresponding remarkable performance on organic synthetic reaction: (a) Total turnover number of r*Aae*UPO (TTN_r_ *_Aae_*_UPO_) of 113,000 for 3 h, (b) TTN_GDH_ of 144,000 for 56 h and (c) TTN*_Ts_*_OYE_ of 1,300 for 7 h. This BPEC device can leverage the merits of high product selectivity and versatility from biocatalysis and photocatalysis, respectively (Figure 12G).

Besides the electrochemical field, thermal energy can also contribute to driving photosynthetic upcycling of solid waste by combining photochemical and thermochemical processes for efficient utilization of the solar light spectrum. The research on photocatalysis is always desired for a reaction system that operates under mild conditions. However, the cleavage of the C─C bond remains a challenge due to its high dissociation energy [289], hence the solar‐driven waste conversion generally needs a lengthy duration to carry out. With this context, the introduction of heat into the reaction can promote the fracture of the C─C bond for the selective conversion of solid waste to fuels or chemicals. Generally speaking, photothermal effects include non‐radiative relaxation and localized surface plasmon effect (LSPR) in semiconductor nanostructures and vibration in molecules [289, 290, 291, 292]. Unlike thermal‐assisted photocatalysis and photo‐assisted thermocatalysis, the coupling of heat and solar energy, which can bring a synergistic effect, has always been used in low‐energy solid waste upcycling. In a typical photocatalytic reaction, most semiconductors respond to the ultraviolet–visible (UV–vis) spectral range but are not activated under the near‐infrared (NIR) spectral range, hence limiting the thermal effect. Herein, the design of photothermal catalysts should consider the effective utilization of the full solar spectrum, vis–NIR light‐to‐heat conversion efficiency, and ameliorated catalytic ability and stability [293]. The introduction of metal‐based catalysts (e.g. Au, Ag, Ru etc.) into the photothermal system can enhance catalytic activity as metal nanoparticles (NP) usually possess a wide light absorption range [294].

**FIGURE 13:**
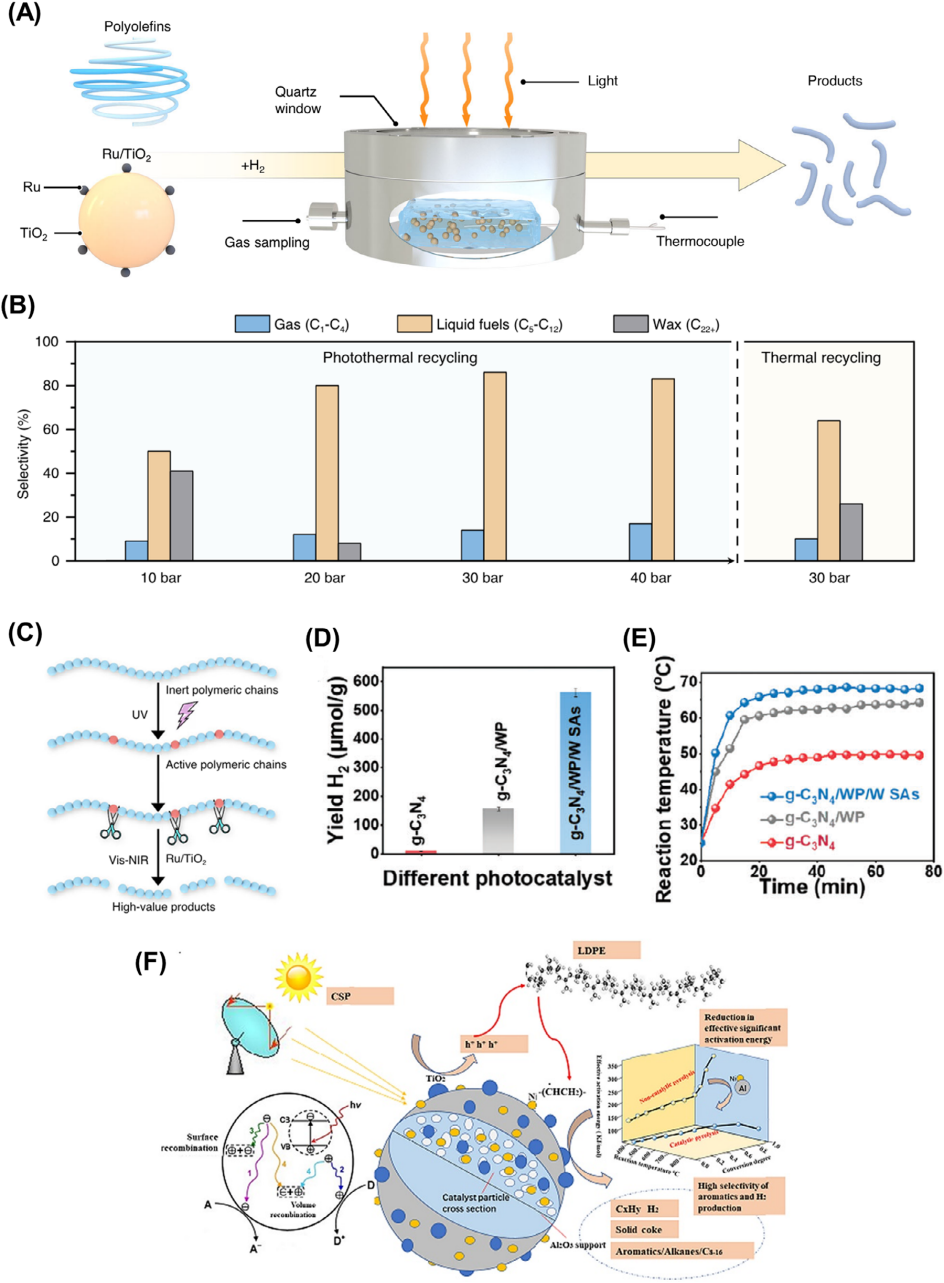
(A) Scheme of photothermal LDPE plastic recycling system, (B) selectivity of product after high‐pressure photothermal recycling of LDPE, (C) proposed LDPE upcycling pathway [295]. Reproduced under the terms of the Creative Commons CC BY license. [295] Copyright 2023, Springer Nature. (D) H_2_ yield via photothermal reforming of PET with different photocatalysts, (E) the solution temperature profile of different photocatalysts [296]. Reproduced under the terms of the Creative Commons CC BY license [296]. Copyright 2024, Wiley‐VCH. (F) Reaction mechanism of recycling LDPE using Ni‐TiO_2_‐γ‐Al_2_O_3_ photothermal catalyst [297]. Reproduced with the permission of Luo et al. [297] Copyright 2022, Elsevier.

For this purpose, Zhang et al. accomplished an efficient photothermal catalytic system with metal NPs to upcycle polyolefin plastic waste into value‐added liquid fuels without using solvent, as shown in Figure 13A [295]. Ru nanoparticles were uniformly dispersed over P25 TiO_2_ semiconductors to form Ru/TiO_2_, realizing the localized heating effect. The catalyst showed high selectivity (>50%) toward C_5_–C_12_ products under concentrated sunlight illumination for 3 h when the system pressure varied from 10 to 40 bar (Figure 13B). Under full‐spectrum Xe lamp irradiation, the light consumed by LSPR could convert to heat energy, resulting in high temperatures in the catalyst, followed by plastic waste melting. With the aid of UV light, the active polymer chain formed reaction sites for cleavage by the Ru NPs, which acted as an active site for C─C and even C─H bond scission to form gaseous and liquid/waxy products (Figure 13C). Beyond the noble metal catalysts, transition metal phosphide and transition metal can be used as cocatalysts and photothermal materials for HER to lower the energy barrier in the photothermal reforming of plastic waste. Zheng et al. combined tungsten phosphide (WP) and tungsten single atoms (W SAs) with porous g‐C_3_N_4_ to leverage the photothermal effect by using the great thermal conductivity of carbon‐based material to recycle waste PET [296]. From Figure 13D, the designed catalyst achieved a promising H_2_ evolution rate of 562.5 µmol g^−1^ h^−1^, which was around 56‐fold higher than that of g‐C_3_N_4_. Without the thermal insulation during the reforming test, the PET hydrolysate temperature of g‐C_3_N_4_‐WP‐W SAs, g‐C_3_N_4_‐WP and pristine g‐C_3_N_4_ increased rapidly from 50.2 to 68.1°C, 44.7 to 62.4°C, and 41.7 to 48.9°C, respectively, under stimulated sunlight (Figure 13E). This result demonstrated that the WP could effectively induce the photothermal effect by plasmonic localized heating, and g‐C_3_N_4_ could serve as an insulator to suppress heat loss from W SAs and WP.

On the other hand, Luo et al. also realized a photothermal catalytic pyrolysis process with temperatures up to 500°C to recycle low‐density PE (LDPE) under simulated sunlight [297]. A Ni‐TiO_2_‐γ‐Al_2_O_3_ photothermal catalyst was used to upgrade the plastic wastes in a H_2_ environment for the generation of valuable aviation fuels, composed of aromatic, diesel, and jet fuel. In fact, Ni‐based catalysts were favorable for the production of value‐added chemicals (e.g. jet fuel and H_2_) under photothermal conditions as Ni was a superior photothermal conversion material. Through tuning the Ni‐TiO_2_ heterojunctions, a wide light absorption range of the catalyst was achieved, which can provide the required heat energy for the upcoming endothermic reaction and enhance the photocatalytic activity of TiO_2_ [297]. The surface heterojunctions of the semiconductors could improve the separation of charge carriers and constrain the recombination rate of electron–hole pairs. The outstanding charge transfer between TiO_2_ and Ni could be attributed to the localized surface plasmon resonance [297, 298]. As shown in Figure 13F, the plausible mechanism for this system could be as follows: (1) the photoexcited electrons migrated from VB to CB, while the photoinduced holes left on VB [299, 300]; (2) LDPE was broken down to long‐chain hydrocarbons with C─H and C─C bonds at high temperature [301]; (3) the intermediates were further oxidized into small molecule hydrocarbons by the holes. Consequently, Ni‐TiO_2_‐γ‐Al_2_O_3_ yielded jet fuel of 80.27% and H_2_ of 34.2 mol kg^−1^ at 500°C. All in all, the introduction of an external field into photocatalysis can enhance the overall performance of the system, which surpasses the result achieved with solely solar energy.

**FIGURE 14:**
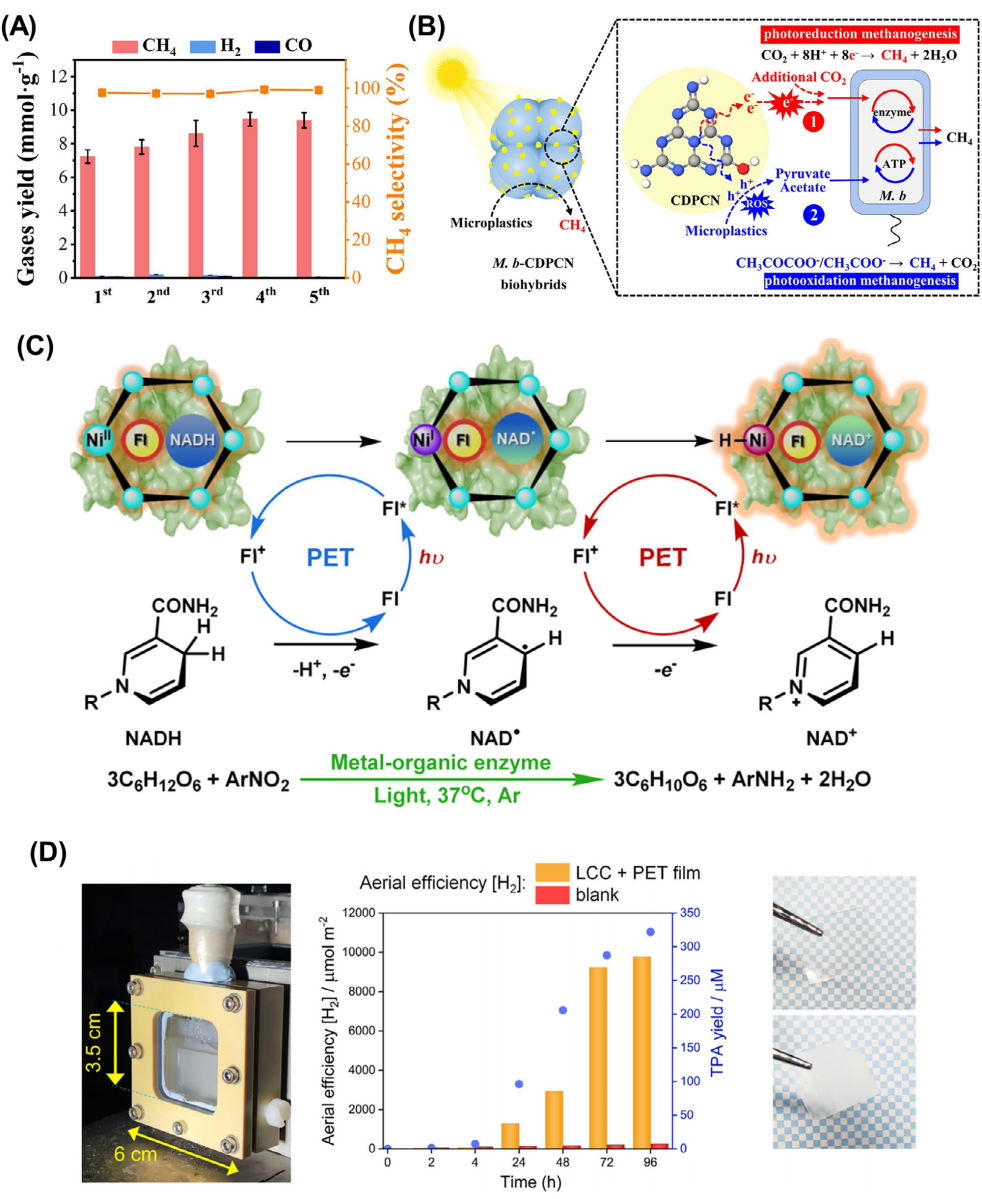
(A) CH_4_ selectivity and average yield in cyclic operation, (B) methanogenesis process using a biotic–abiotic hybrid [306]. Reproduced with the permission of Ye et al. [306] Copyright 2022, Wiley‐VCH. (C) Consecutive reactions of cofactor regeneration [307]. Reproduced with the permission of Li et al. [307] Copyright 2024, American Chemical Society. (D) Integrated system with LCC enzyme and PET film for photoreforming reaction [308]. Reproduced under the terms of the Creative Commons CC BY license [308]. Copyright 2023, American Chemical Society.

In the last couple of years, biocatalysis has been recognized as a green technique in organic synthesis due to its mild and environmentally benign reaction conditions [302, 303]. In particular, the advances in directed evolution allowed the unsophisticated modification of enzymes for adapting to different reaction conditions, in which engineering the enzyme can enable it to withstand harsh temperatures and pH values and react to synthetic substances for the production of new products [304, 305]. In the same period time, artificial photosynthesis has also been studied and developed extensively into a highly investigated sustainable approach. With this context, a great attempt has been made to integrate both green technologies to take advantage of photocatalysis (i.e. wide applications and use of renewables) and biocatalysis (i.e. high selectivity and efficiency) for building a biotic–abiotic hybrid system. For example, Ye et al. reported a solar‐driven biohybrid system for the conversion of plastic waste to methane by combining *Methanosarcina barkeri (M.b*) and carbon dot‐functionalized polymeric carbon nitride (CDPCN) [306]. The use of methanogenesis with appropriate redox ability resulted in a remarkable increase in methane production and selectivity from pretreated PLA (Figure 14A). The reaction mechanism of such a system involves two processes for the reforming of PLA into methane: (i) photoreduction of CO_2_ and (ii) plastic oxidation, as shown in Figure 14B. The UV light irradiation induced the electron–hole pairs in CDPCN, where the electrons travelled to CB followed by *M.b*. The photoexcited electrons were used as reducing equivalents by M.b with the help of an additional CO_2_ source from NaHCO_3_ to generate methane. Meanwhile, the PLA monomers underwent oxidation by reactive oxygen species to produce intermediates, such as pyruvic acid and acetic acid. Then, they could be utilized as potential carbon resources for methanogenesis to produce CH_4_. This study provides a unique insight into the use of enzymes for the conversion of plastic waste into methane with promising selectivity.

Apart from that, Li and his team developed an approach to merge a photocatalytic system with enzymatic transformation by integrating a metal organic cage with glucose dehydrogenase to upcycle biomass via tandem enzymatic NADH regeneration (Figure 14C) [307]. Under light irradiation, the cage‐fluorescein‐NADH clathrate served as a photoactive relay to carry out two photoinduced electron‐transport reaction followed by oxidation of NADH to NAD^+^. The dehydrogenase would extract a hydride from glucose to generate gluconolactone and replenish NAD^+^. This integrated system resulted in high catalytic activity, achieving 226 µL of hydrogen yield and 16.8 µmol of gluconolactone‐derived products. The grafting enzyme also enabled the tandem nitrobenzene reduction with a TTN of 15,000, offering an innovative biotic–abiotic hybrid system to produce solar fuels and reform biomass. In previous reported studies, acid or alkaline pretreatment is used to facilitate the hydrolysis of polymer into their monomers, however such pretreatment conditions violate the concept of Green Chem. As such, Reisner et al. developed a chemoenzymatic photoreforming system by integrating the enzymatic degradation of synthetic PET plastic with photoreforming to produce H_2_ in a neutral environment [308]. As shown in Figure 14D, the enzymatic hydrolysis of plastic and photocatalytic H_2_ evolution were conducted simultaneously in a self‐designed photoreactor for catalyst recovery and continuous flow process. The hydrogen evolution rate and terephthalic acid yield reached 9.784 mmol m^−2^ and ∼322 µm after 96 h. The color change in PET film indicated that the enzymes depolymerized PET to ethylene glycol and terephthalic acid. This chemoenzymatic pretreatment for plastic photoreforming enabled an alkali‐free depolymerization pretreatment process and the generation of fuel in a continuous flow process.

In general, the separation and mitigation of photoinduced carriers always play crucial role in a typical photocatalytic reaction. To suppress the recombination of photogenerated charge carriers, the construction of internal electric field inside photocatalysts can provide sufficient driving force to separate electron–hole pairs. Thus, the pyroelectric and piezoelectric built‐in electric field can be introduced into the photocatalysts to develop a nanostructure composite that can bring synergistic effect for ameliorating solar‐to‐energy conversion. Of note, the pyroelectricity and piezoelectricity could be coupled effectively, as evidenced by the several studies. For instance, Wei and his coworkers prepared a PVDF/Fe_3_O_4_@g‐C_3_N_4_ (PVFC) micromotors to nurture the piezoelectric and pyroelectric effects simultaneously to improve the directional migration of charge carriers for biomass reforming [309]. With the assistance of both effects, the H_2_ generation rate of PVFC micromotors was 42.3 µmol h^−1^, exceeding the photocatalytic water splitting reaction by 31.9 times. Moreover, the apparent quantum yield reached 12.6% under full spectrum solar light illumination, showcasing that great synergy of both electric field in this micromotor. As discussed in the above section, there is only one study using the piezoelectric effect for plastic photodegradation [107], and to the best of our knowledge, limited study was observed in applying such an external field in plastic photoreforming.

By incorporating an external field into the photocatalytic system, the activity of the photocatalyst can be effectively ameliorated by hampering the charge carrier recombination and lowering the energy barrier. Notably, a variety of solar fuels has also been observed to produce with the introduction of different fields, widening the versatility and practicability of multi‐energy integrated photocatalysis in solid waste upcycling. All in all, the synergy between two or even three fields allowed the simultaneous contributions for effective utilization of solar energy, enhancement of selective product generation, and improve catalytic stability. However, identifying the precise mechanistic pathway of multi‐energy integrated photocatalysis systems is a formidable task. Thus, in‐situ characterization analysis and computational science should be coupled with experimental results to explore and abstract the in‐depth mechanisms underneath multi‐field photocatalytic processes.

## 5 Conclusion & Future Recommendations

Collectively, solid waste signified an inexpensive, abundant, carbon‐ and hydrogen‐rich feedstock to address the alarming planetary crisis of fossil fuel depletion and growing energy demand. However, with its technological maturity, the conventional thermochemical route may still be the primary way to reform solid waste for an extended period. To reduce and prevent the use of thermal transformation of solid waste, the management strategy of solid waste necessitates an innovative and sustainable approach that can eradicate the risk of degrading the environment and establish a new paradigm toward a circular economic model. Hence, throughout this review, the concept of solid waste valorization via artificial photosynthesis has been presented as “the use of solid wastes as a feedstock for the generation of solar fuels such as H_2_, hydrocarbons, and valuable chemicals” to bear a carbon circular economy in mind. This review provides an outline of the latest progress in solar‐driven solid waste conversion, with the purpose of shining light on the novel photocatalyst design (e.g. morphological alteration, defect engineering, heterojunction construction, and heteroatom doping) and fundamental reaction pathway of solid waste upcycling. Overall, the modification strategies for photocatalysts are intended to engineer physicochemical properties, including band gap alignment, catalytic active sites, electronic properties etc., for ameliorating photocatalytic performance. By harvesting the synergistic effects toward improved solar‐to‐chemical reactions, the upcycling of solid waste can be facilitated with the property‐activity relationship between catalysts and solid waste.

Globally, plastic and biomass photoreforming technology can bring economic merits and sustainability beyond water splitting, contributing to achieving sustainable development goals (SDGs), particularly SDG 6 (Clean Water and Sanitation), SDG 7 (Affordable and Clean Energy), and SDG 12 (Responsible Consumption and Production). As such, photocatalytic upgrading of solid waste enables the creation of a new energy system and circular waste economy to light up the future towards a greener society. Considering the perspective of the trinity of sustainability, plastic and biomass upgrading offers remarkable significance and societal influences as the world grapples with solid waste and energy transitions. Herein, this technology opens up potentials of (1) producing clean fuel from waste, (2) alleviating the waste crisis worldwide, (3) establishing a new value chain from waste, (4) reducing the cost of hydrogen production using a gratis feedstock, (5) changing public sentience of biomass and plastic from waste to resource, and (6) encouraging public awareness of waste‐to‐wealth model. In short, this innovative technology aims to sustainably address the solid waste crisis by ensuring economic development, environmental protection, and societal well‐being. Gratifyingly, it is possible to envision that the ongoing advancements in solid waste conversion could play an essential role in transitioning to a more eco‐friendly and sustainable future.

However, this technology is still in the infancy phase owing to ample room for enhancement in photocatalytic reaction efficiency, product selectivity, stability, and economic viability. Therefore, some challenges are presented as follows: (1) low conversion efficiency and total energy efficacy of solid waste photoreforming that result in restraining its practicability to a larger scale, (2) limited discovery and research on other photocatalysts where only a few kinds of semiconductors (e.g. g‐C_3_N_4_, TiO_2_, CdS, ZnIn_2_S_4_) focused on existing studies, (3) deficient knowledge of the optimization of catalyst materials and components which is important in improving performance, stability, selectivity and feasibility, (4) ambiguous reaction mechanism leads to uncontrolled activity and selectivity of the products and (5) insufficient study on improving the reaction conditions. In the context of photocatalytic solid waste conversion, qualitative and quantitative analyses serve as an important tool to identify unknown products from different solid waste and target the desired products, respectively. Moreover, photodegradation or overoxidation of solid waste into CO_2_ could contribute to the carbon footprint, resulting in environmental degradation.

Continual research and innovation that involves interdisciplinary studies are required to address these challenges. As such, several research directions for future solid waste photoreforming that are worthwhile conducting further investigation are highlighted below:

1. Design of photocatalyst: Developing cost‐effective, highly efficient, and durable photocatalysts to upcycle solid waste into value‐added products with high selectivity is crucial for upscaling to commercialization. Due to the rapid recombination of electron–hole pairs and undesirable utilization of the full solar spectrum, the disadvantageous solar‐to‐chemical conversion efficiency becomes the primary factor that impedes practical application on a larger scale. Additionally, only finite types of photocatalysts, such as TiO_2_, CdS, g‐C_3_N_4_, and ZnCdS, are reported to date in traceable studies, and many other semiconductors have not been exploited until now, thus restricting the development of solid waste photoreforming. In fact, extensive research works of photocatalytic water splitting and CO_2_ reduction could be the inspiration and serve as a diverse library to design an efficient photocatalytic waste conversion system for optimizing the catalyst material and alleviating the reliance on noble metal catalysts. Recently, the application of single atom, metal alloy, and spinel oxide in electrocatalysis also can be a reference for promoting the performance of photoreforming by maximizing the charge separation efficiency [315, 316, 317]. Several modification strategies for development of parent catalysts and composite catalysts, such as heterojunction construction, introduction of heteroatom, structural alteration, band gap positioning, defect engineering, and so forth, have provided tremendous opportunities for bolstering and boosting the solar‐driven solid waste upcycling. In particular, semiconductor with narrow band gap can harvest visible and infrared light to promote solid waste conversion efficiency. Furthermore, the introduction of quantum dots and plasmonic nanoparticles into the photocatalysts can help to broaden the absorption range of light to improve photocatalytic reaction performance.
2. In‐depth mechanistic study and computational modeling: A comprehensive understanding of the reaction mechanisms in solid waste photoreforming is a tedious and time‐consuming task involving microchemistry and catalysis. However, this endeavor is essential in the quest to design and optimize catalysts for the selective generation of products, outstanding durability, and superb catalytic activity. Taking the overoxidation of solid waste as an example, modifying the surface charge and structure of catalysts can improve the interaction between reactants and catalysts (i.e. adsorption and desorption) to achieve partial solid waste oxidation process and promising H_2_ evolution rate at the same time to avoid production of CO_2_. To this end, pairing the advanced characterization techniques with computational modeling is desirable to provide pivotal insights for elucidating the full mechanistic underlying the plastic reforming. Multitudinous in situ/operando characterization methods, such as in situ electron paramagnetic resonance (EPR) spectroscopy, in situ FTIR spectroscopy, in situ Raman spectroscopy, and in situ X‐ray absorption spectroscopy, are used to monitor the evolution of reactants, intermediate, and products during the reaction, thus providing key understanding of the dynamic behaviors of catalytic active sites toward targeted products. On top of that, NMR, GC‐MS, and ultra‐high performance liquid chromatography (UHPLC) can also be used to precisely detect and quantify the presence of intermediates and products to better elucidate the reaction pathway. Of particular note to the organic transformations, the bond scission for selective photocatalytic synthesis of product that has been realized can also deliver invaluable insight to solid waste transformation [22, 24, 31, 32]. Furthermore, the isotope labeling test can also be performed in plastic and biomass valorization to provide more mechanistic insights on the identification of reaction intermediates and verification of the origin of evolved products. For example, the replacement of proton to deuterium can unveil that the source of hydrogen originates from water splitting rather than plastic and biomass. Computational modeling such as density functional theory (DFT) can be used to accompany experimental analysis by simulating the dynamic evolution of the reaction to shed light on the reaction mechanism. In addition, as Industrial Revolution 4.0 came by, artificial intelligence (AI) or machine learning (ML) can be applied to estimate the structural properties of catalysts such as running DFT calculations of Fermi energy and Gibbs free energy. Furthermore, with the aid of AI/ML, unearthing novel materials can be accelerated for future viable industrial operations by integrating theoretical results and past studies in plastic and biomass upgrading processes and embedding the scientific knowledge (e.g. physicochemical properties, charge dynamics, and environmental context) in feature space for the development of predictive models. For example, machine learning was utilized to integrate with DFT for predicting the photocatalytic C─C bond cleavage in lignin [318]. Hence, the application of AI not only improves the efficiency of discovering new catalysts but also provides a comprehensive understanding of photocatalytic mechanisms, thereby contributing to the development of highly effective photocatalytic materials.
3. Solid waste categorization and pretreatment: Despite biomass and plastic discussed in this review, there are all sorts of solid waste (e.g. food, paper, rubber, glass etc.) produced daily worldwide, where all these wastes should be collected and sorted accordingly. Up to now, most of the current studies focused on the conversion of simple monomers of biomass and analytical grade of plastic to selectively generate desired products. However, there are various impurities and additives presented in real‐life solid waste, in which the final products in the conversion of those wastes may be deviated from lab‐experiment scale. The reaction intermediates from unknown substances may create possible reaction pathways for inhibiting the generation of desirable products, thus reducing the overall value of products. Hence, more effort should be paid to categorize real‐life solid waste in a systematic way for larger scale implementation. Different from traditional automated systems that differentiate materials based physicochemical properties, smart automation with artificial intelligence is a powerful tool to help identify and sort the solid waste effectively based on machine learning algorithms [319]. To achieve high conversion efficiency of raw materials under mild conditions, substantial endeavors should invest to develop a milder, low‐cost, and eco‐friendly pretreatment method to replace the alkaline pretreatment for a better ecological system. Green pretreatment strategies, such as enzymatic pretreatment, bio‐based solvent, and mechanical degradation, can be incorporated into processing solid waste instead of chemical pretreatment. For example, solar heat treatment can be applied as green thermal treatment to treat solid waste. Consequently, the reaction conditions, including reactant feed ratio, light intensity, photocatalyst loading etc., should be optimized to achieve higher yield and selectivity.
4. Development of photoreactor: Moving on to the industrial implementation of artificial photosynthesis technology, a photoreactor is important to effectively utilize solar energy for the interactive contact between photocatalyst and reactant, leading to the collection of resultant products. In lab‐scale experiments, existing photocatalytic reactions for solid waste valorization mostly rely on a powder suspension system, which is unfavorably viable on a larger scale. Therefore, developing a photoreactor is another key research area that should not be trivialized in this field. Firstly, before designing the reactor, the immobilization of photocatalyst powder on a substrate to form a thin film is an inexpensive and feasible approach for benefiting catalyst reuse and realizing the continuous flow process. The thin catalyst films were applied for constructing flat panel reactors in the existing system. For example, Nishiyama et al. reported a 100 m^2^ array of panel reactors for hydrogen generation with a 0.76% solar‐to‐hydrogen efficiency [320]. Meanwhile, parabolic trough reactors are another commonly used configuration in photocatalytic applications. This unique model offers concentrated sunlight irradiation to the reactor that is placed along the focal line of the parabolic [321, 322]. Nonetheless, there are shortcomings existing in these two systems: (1) In panel flow reactors, inadequate adsorption and desorption between catalyst film and reactants may result in reducing the overall performance compared to particulate photocatalysts, and (2) in parabolic concentrators, only direct sunlight or light that is reflected at a certain angle will be entered to the transparent tube with indefinite efficiency. With this context, the design of a panel photoreactor should consider four factors: (a) light utilization and distribution, (b) photon transmittance, (c) mass transfer, and (d) scalability and versatility. Moreover, the light orientation, design of the reactor tube, and parabolic geometry should be involved in the development of a concentrating reactor to ensure efficient light capture and effective photocatalyst exposure.

Despite the breakthroughs that have been made in recent years, there is still a long way to go in the realm of photocatalytic solid waste conversion. To uncover the hidden potential of solar‐driven upcycling of solid waste into valuable products, multidisciplinary field of study (i.e. chemistry, physics, catalysis, material science, reaction engineering, and computational modeling) should be pursued cooperatively for driving the commercialization of this technology. As a final note, we hope that solid waste photoreforming can be extensively established in the future to achieve a carbon circular economy with the aid of collective achievements by the researchers.

## Conflicts of Interest

The authors declare no conflicts of interest.

## Data Availability

Data sharing is not applicable to this article as no new data were created or analyzed in this study.
